# Structural features embedded in G protein-coupled receptor co-crystal structures are key to their success in virtual screening

**DOI:** 10.1371/journal.pone.0174719

**Published:** 2017-04-05

**Authors:** Thomas Coudrat, Arthur Christopoulos, Patrick Michael Sexton, Denise Wootten

**Affiliations:** Drug Discovery Biology, Monash Institute of Pharmaceutical Sciences and Department of Pharmacology, Monash University, Royal Parade, Parkville, Victoria, Australia; Institute of Materials Science, GERMANY

## Abstract

Structure based drug discovery on GPCRs harness atomic detail X-ray binding pockets and large libraries of potential drug lead candidates in virtual screening (VS) to identify novel lead candidates. Relatively small conformational differences between such binding pockets can be critical to the success of VS. Retrospective VS on GPCR/ligand co-crystal structures revealed stark differences in the ability of different structures to identify known ligands, despite being co-crystallized with the same ligand. When using the OpenEye toolkit and the ICM modeling package, we identify criteria associated with the predictive power of binding pockets in VS that consists of a combination of ligand/receptor interaction pattern and predicted ligand/receptor interaction strength. These findings can guide the selection and refinement of GPCR binding pockets for use in SBDD programs and may also provide a potential framework for evaluating the ability of computational GPCR binding pocket refinement tools in improving the predictive power of binding pockets.

## Introduction

G protein-coupled receptors (GPCRs) form the largest protein superfamily in mammalian genomes [1,2] and play a crucial role in physiological processes through mediating the cell’s response to extracellular signals [3]. Among the members of this family are receptors for hormones, neurotransmitters, small peptides and even photons of light [4]. Their implication in various pathophysiologies has made them attractive targets, with over 40% of currently marketed drugs targeting this family of proteins [5]. GPCRs can be selectively activated and inhibited via their extracellular face by endogenous agonists and inhibitors, respectively. Dysregulation of this finely tuned machinery is a common cause of pathology that can be alleviated by intervention with synthetic ligands acting at GPCRs to recover normal function [6].

Design of novel small molecule chemical compounds that target a specific GPCR with high affinity and selectivity is challenging. Lead compounds have been identified in the past largely through high-throughput screens (HTS), where a physical assay is used to rank a large library of compounds to identify chemical scaffolds that can be optimized. Running an HTS on several million compounds is expensive, and in recent years there has been a growing interest in computational methods that help focus the physical screen on a subset of molecules predicted to bind the target of interest. These can be divided into ligand-based drug discovery (LBDD) and structure-based drug discovery (SBDD) methods. LBDD methods link the physicochemical properties of known active molecules with their measured activity on the GPCR target, whereas SBDD methods can be readily applied to a new GPCR target for which there is limited ligand data. As they rely on the 3D structure of the target protein, SBDD methods offer better potential for identification of novel ligand scaffolds through virtual screening (VS).

SBDD requires detailed understanding of the molecular interactions between a ligand and its receptor. Ligand docking is a computationally cost-effective method that predicts the conformation of a ligand inside the binding pocket of the target protein, based on the physicochemical properties of both the ligand and the target. VS by docking ranks libraries of small molecules based on a docking score, which is followed by experimental validation of the top ranked virtual hits predicted to be enriched in active compounds [7]. VS has extensively and successfully been used on many soluble protein drug targets (e.g. enzymes) and more recently on GPCRs [8].

The increased success in recent years for GPCR SBDD is in part due to experimental breakthroughs in GPCR X-ray crystallography, opening up the GPCR structural landscape at the atomic level. Through the GPCR X-ray crystallography revolution, a total of 154 GPCR structures have been obtained, including 38 unique structures, providing atomic details on the arrangement of their seven transmembrane (7TM) helices [9]. Additionally, 73 unique ligand/receptor complexes provide critical information on ligand interaction patterns, including differences between agonists and inhibitors. Resolution of these crystal structures is a metric that is often used to evaluate the quality of the overall X-ray crystal structure with a higher resolution guiding greater accuracy of the position of atoms in the resulting model. Further assessment of fit between the experimental data, the electron density map, and the generated model of the co-crystal structure, can be performed on a residue per residue basis using real-space correlation coefficients (RSCCs).

The choice of GPCR structure for use in SBDD is critical for the outcome of the SBDD program. Indeed, even small conformational changes in a binding pocket, induced by the stabilizing ligand can have a marked effect on VS results as observed in studies where GPCR X-ray crystal structures stabilized by a ligand of a given pharmacology preferentially select new compounds with that same pharmacology (i.e. agonist vs inhibitor) [10,11]. In these cases, ligand/protein interaction fingerprints (IFPs) may be useful to shift the selectivity of a crystal structure in VS towards that of a different pharmacology, if the desired IFP is known [11]. This was exemplified in a recent case study using β-adrenoceptor crystal structures where the predicted IFP for a full agonist was successful at screening for agonists over antagonists in VS performed on crystal structures co-crystalized with either full agonists, partial agonists, antagonists or inverse agonists [11]. However, despite this success, the selected crystal structure still influenced the extent to which the IFP was able to shift the selectivity and the final enrichment values within these screens.

Furthermore, two co-crystal structures of the same GPCR bound by the same small molecule ligand do not always have identical binding pocket shapes and ligand/receptor interaction patterns [11,12]. This is most clearly exemplified when looking at the different orientations of ZM-241385 (ZM) in the adenosine A_2A_ receptor (AA2AR) binding pockets, that may arise due to different protein engineering methods, such as insertions and/or thermostabilizing mutations [13] or different crystallization conditions, for example, differences in pH [14]. These can lead to subtle distinctions in receptor side-chains and ligand conformation, in turn resulting in different IFPs in the co-crystal structure. There are now several GPCR structures available that are bound to the same ligand, offering an important view of the variability that can occur between GPCR X-ray structures. These structures can be grouped based on their bound ligand (Table 1).

**Table 1:** GPCR X-ray crystal structures co-crystallized with a common orthosteric ligand.

GPCR	PDB ID and chain(s) used	Orthosteric ligand name and abbreviation	Resolution (Å)	Stabilization					References
				Ligand pharmacology	Other molecules	Insertion	Missing side-chain(s)	Mutation(s)	
ADORA2A	4UG2 (A, B)	CGS-21680 (CGS)	2.60	selective agonist	dimer	-	chain A: R222, S223. chain B: E219, R220, R222, S223	L48A, A54L, T65A, **Q89A**	Lebon et al. 2015 [15]
	4UHR		2.60		-	-	L216, E219	L48A, A54L, T65A, **Q89A**	Lebon et al. 2015 [15]
	3EML	ZM-241,385 (ZM)	2.6	high-affinity subtype selective antagonist	-	T4L (ICL3)	-	-	Jaakola et al. 2008 [16]
	3PWH		3.3		-	-	-	A54L, T88A, R107A, K122A, L202A, L235A, V239A, S277A	Doré et al. 2011 [13]
	3VG9		2.70		antibody Fab 2838	-	-	N154Q	Hino et al. 2012 [17]
	3VGA		3.10		antibody Fab 2838	-	R220, R222	N154Q	Hino et al. 2012 [17]
	4EIY		1.8		-	BRIL (ICL3)	Q148, E161, R220, R293	M215W, H310I, R314L	Liu et al. 2012 [18]
ADRB1	2Y00 (A, B)	Dobutamine (DOB)	2.50	agonist	dimer	-	-	R68S, M90V, C116L, Y227A, A282L, F327A, F338M, C358A	Warne et al. 2011 [14]
	2Y01 (A, B)		2.60		dimer	-	chain A: L83	R68S, M90V, C116L, Y227A, A282L, F327A, F338M, C358A	Warne et al. 2011 [14]
	2VT4 (B)	Cyanopindolol (CYP)	2.70	weak partial agonist	tetramer	-	-	R68S, M90V, C116L, Y227A, A282L, F327A, F338M, C358A	Warne et al. 2008 [19]
	2YCX (A)		3.25		dimer	-	-	R68S, M90V, C116L, Y227A, A282L, F327A, F338M, C358A	Moukhametzianov et al. 2011 [20]
	2YCY (A)		3.15		dimer	-	-	R68S, M90V, C116L, Y227A, A282L, F327A, F338M, C358A	Moukhametzianov et al. 2011 [20]
	4BVN		2.10		-	-	-	R68S, M90V, C116L, I129V, Y227A, A282L, D322K, F327A, F338M, Y343L, C358A	Miller-Gallacher et al. 2014 [21]
ADRB2	3P0G	BI-167107 (BI)	3.5	agonist	Camelid antibody fragment: nanobody Nb80	-	-	E27Q, N187E	Rasmussen, Choi, et al. 2011 [22]
	3SN6		3.2		Heterotrimeric Gs protein. Nanobody Nb35.	T4L (N-term)	R63, K97, T98, W99, F101, K149, R175, D192, **F193, F194, T195**, K267, H269, K270, L272, Q299, N301, L302, R304, E306, R333	M96T, M98T, N187E	Rasmussen, DeVree, et al. 2011 [23]
	4LDE		2.79		Nanobody Nb6B9	T4L (N-term)	K60, E62, K149, F223, Q224, Q231, K263, F264, K270	M96T, M98T, N187E, C265A	Ring et al. 2013 [24]
	2RH1	Carazolol (CAR)	2.40	inhibitor	-	T4L (ICL3)	D29	N187E	Cherezov et al. 2007 [25]
	4GBR		3.99		-	T4L (N-term)	-	M96T, M98T, N187E	Zou, Weis, and Kobilka 2012 [26]
DOR	4EJ4	Naltrindole (NAL)	3.4	inhibitor	-	T4L (ICL3)	K79, V154, K155, R192, Q201, R241, S242, R244, K252, R291, R292, E323, N324, R327	M80L, R190Q, D290N (*)	Granier et al. 2012 [27]
	4N6H		1.8		-	BRIL (N-term)	R41, V154, L157, K166, R192, D193, K250, E251, K252, R254, K326, R330, Q331, R334,K335	P37S	Fenalti et al. 2014 [28]

For each structure, the name of the GPCR, the PDB ID accession number, the name of the bound orthosteric ligand and the resolution of the structure are listed. Further information relating to the components contributing to the stabilization of the structure are also included; these include details on the ligand pharmacology and selectivity, information about molecules used for receptor stabilization as well as notes about dimer state where relevant, insertion molecule (and position of the insertion), residues for which at least one heavy-atom side-chain is missing from the structure and mutations present in 7TM domain structure (residues in bold refer to residues in the binding pocket: combination of residues within 5 Å of bound ligands). References to each X-ray crystal structure are also provided. (* residues different compared to the *human* DOR gene).

The increased accessibility of GPCR structures is paired with the availability of annotated databases of compounds that are known to be active on these GPCRs. Compound databases with linked GPCR activity such as ChEMBL [29], ZINC [30], PubChem [31], GLIDA [32], IUPHAR [33] are of crucial importance in the early stage of a SBDD program. The capacity of GPCR binding pockets to assign high docking scores to known active compounds is one of the metrics that evaluate these binding pockets before prospective VS. Furthermore, developing decoy libraries that have similar physicochemical properties to the known compounds, but are not validated ligands of a particular GPCR, allows the evaluation of GPCR binding pockets for their capacity to recover known ligands over decoys. The GPCR ligand library and GPCR decoys database (GLL/GDD) was generated to run such retrospective VS, where for each known ligand there is a set of 39 decoy ligands matching its physical properties [34].

Another important consideration for GPCR SBDD programs revolves around the validation of a GPCR structure that relies known ligands. Small molecules often have more than one chiral center, leading to multiple combinations of molecules with identical composition but different geometries or enantiomers. Chiral molecules are often not tested in biological assays as pure enantiomeric forms, but rather in a racemic mix containing all possible enantiomers. Hence when a compound’s pharmacology is identified, the exact active enantiomer is often not known, and therefore the activity is attributed to the racemic mix. However, the enantiomeric state can have a drastic effect on activity, for example, between the identification of an active allosteric modulator and inactive compound at the muscarinic acetylcholine receptor 5 [35] or two different biased agonists at the β-2 adrenoceptor (B2AR) [36]. Properly recording and dealing with enantiomeric states is therefore an important consideration in GPCR SBDD programs.

In this study, we sought to establish criteria that could be used to guide the selection of a crystal structure for use in VS, when more than one structure is available. Seven GPCR/ligand co-structures, where there is more than one structure crystallized with the same ligand, were selected and evaluated using VS, ranking known ligands relative to decoys and known agonists relative to inhibitors. Using the Molsoft ICM modeling software [37] for docking, we report stark differences between the performance of GPCRs co-crystallized with the same ligand, for known ligand enrichment, as well as selectivity for agonists or inhibitors. Furthermore, using the ICM modeling software [37] and the OpenEye OEChem toolkit [38], we extracted structural and conformational information about each crystal structure and identified key criteria that can influence the predictive power of binding pockets. These include a combination of ligand/receptor interaction patterns and predicted interaction strength in the co-crystal X-ray structure used for screening. These findings can guide the selection and refinement of binding pockets for use in GPCR SBDD programs.

## Methods

### X-Ray co-crystal binding pocket selection and preparation

We selected seven GPCR co-crystal structures for which the bound ligand is reported in at least two GPCR co-crystal structures. The selected structures feature three agonist-bound and four inhibitor-bound GPCRs including AA2AR, β-1 adrenoceptor (B1AR), B2AR and δ-opioid receptor (DOR) [13–28], all class A GPCRs. Details regarding the resolution, protein engineering methods used for stabilization as well as mutated or missing residues are listed in Table 1. In this study, we investigated these ligand/receptor complexes and how small differences between co-crystal structures could influence VS performance. In structures where crystal symmetry yielded oligomers, the representative monomer described for that X-ray structure was used (2VT4 (B chain), 2YCX (A chain) and 2YCY (A chain)). In cases where different IFPs were observed amongst monomers, each monomer was screened as a separate binding pocket, and the chain was used as a suffix: 4UG2-A and 4UG2-B, 2Y00-A and 2Y00-B, 2Y01-A and 2Y01-B.

Mutations introduced for crystallography and missing side-chains present within 5 Å radius of the bound ligand are critical to small molecule docking and in turn could affect comparisons of VS performance between GPCR co-crystals. These are shown in Table 1. In the case of AA2AR CGS-21680-bound (CGS) structures, all three binding pockets contain the same mutation, in which case comparison is valid. Among AA2AR ZM-bound binding pockets, 3PWH was crystallized making extensive use of thermostabilizing mutations. This includes only one mutation within 5 Å of ZM, T88A. In this case, no polar contact is made between threonine 88 and ZM in other pockets and its position deep inside the binding pocket makes it unlikely to influence docking experiments. Finally, missing side-chains in the B2AR BI-bound 3SN6 were added using the ICM software and were optimized in presence of BI. This optimization protocol was used on all B2AR BI-bound pockets.

GPCR models were prepared from the deposited PDB files as follows: (i) non-GPCR residues, and non-ligand molecules (including waters) were removed. (ii) GPCR residues were renumbered in cases where the numbering didn’t match the gene numbering (4LDE and 4GBR) (iii) Ligand/receptor complex was converted to an ICM object that adds hydrogen atoms and missing side-chains and flips the asparagine, glutamine and histidine side-chains to improve molecular interactions. The conversion to an ICM object is a necessary step for energy calculations performed on the molecular model during docking. This optimization did not have a notable impact on VS performance in all cases tested (S1 Fig), but did have a significant impact on the conformation of the side-chains added by ICM when these were missing from their original X-ray structure. Therefore, it was applied on B2AR BI-bound pockets as described above. Waters are removed as X-ray structures with a range of resolution are compared, most with resolution too low to identify water positions. Although binding pocket waters are important for docking when their positions are known, this is rarely the case for GPCR X-ray structures, thus deleting waters from all binding pockets enables an even comparison on the binding pockets’ VS performance.

### Ligand libraries

Ligand libraries were selected from the GPCR ligand library and GPCR decoys database (GLL & GDD) [34]. The co-crystallized ligand of each GPCR complex used in this study were added to their corresponding GLLs, and the lists were manually inspected and errors removed. The ICM software was used to manage the GLL and GDD library, which were downloaded and processed in SDF format. These libraries, hereafter termed the original GLL/GDD, store a single 3D enantiomer to represent each ligand. We identified these libraries in many instances contained the wrong enantiomer for known ligands and therefore may also contain the wrong enantiomer for ligands where the enantiomeric state is unknown. We decided to sample and dock all enantiomeric forms for each ligand in the GLL/GDD. We thus used ICM to generate a new library, hereafter termed the racemic GLL/GDD, which involved converting the original GLL/GDD to 2D depiction and assigning racemic flags to the molecules containing at least one chiral center. By docking all enantiomeric states for each racemic mix, we ensure that the right enantiomer is evaluated. In some cases, this library processing generated doubles in the racemic GLL/GDD, as some molecules in the original GLL/GDD were present in more than one enantiomeric state. These molecules were counted only once in the VS analysis. We used the racemic GLL/GDD to make observations and draw conclusions, but we also screened all binding pockets with the original GLL/GDD for comparison (S2 Fig).

Each known ligand library was clustered based on chemotype similarity and a maximum of 4 clusters (A, B, C and D) were selected to illustrate the chemotype variety of the library, while the cluster ‘other’ was populated with the remaining molecules. We opted to analyze known ligand diversity based on a set number of clusters instead of a set cutoff value in order to generate a manageable number of clusters for analysis. As resulting cutoffs vary amongst different libraries, these are only used to comment on chemotype preference within a library and are not intended to be used for comparison amongst libraries. Clusters containing the X-ray ligand were annotated with its three letter ligand name, and the center of each chemotype cluster is identified with an asterisk (S3, S4, S5 and S6 Figs). The center of each cluster was used to illustrate the chemotype of that cluster, and the chiral center composition of each chemotype was calculated (S1 Table).

### Virtual screening setup

VS was performed using ICM version 3.7-3b. The docking box was defined by selecting all residues within 4 Å of the crystal ligand, which was subsequently removed from the model. Each ligand from the library was docked three times. VS parameters were left as default except for the following parameters that were modified to include all screened molecules: maximum number of hydrogen bond donor, set to 15; maximum ligand size, set to 1000 (calculated as 15 x number of heavy atoms); maximum number of hydrogen bond acceptor/donor, set to 20; maximum predicted logP value set to 15; minimum predicted logP value, set to -10. Additional parameters identified in the laboratory to improve VS outcome were modified, including ring sampling parameter set to 1, charge mode of ligand ionizable groups set to auto. The effort value influences the length of the docking simulation and was set to 5 throughout this study, unless stated otherwise. Finally, the racemic GLL/GDD was screened using the following parameters: database type set to “mol 2D” and racemic sampling set to “yes”. The latter performs a sampling of all enantiomers for that ligand, all of which are docked, and the best scoring enantiomer is returned as a result.

### Virtual screening analysis

Docking of each ligand involves a conformational sampling performed by a biased probability Monte Carlo procedure [39]. Given the stochastic nature of this procedure, docking was repeated three times and the best scoring repeat was retained to represent that ligand in terms of conformation and docking score. VS results were visualized using receiver operating characteristic (ROC) curves, which show the percentage of true positives as a function of the percentage false positives recovered in the VS ranked ligand library. Two types of ROC curves were used to discriminate (i) known ligands against decoys, and (ii) agonists against inhibitors (or vice versa). Binding pockets were compared by their capacity to rank true positives higher relative to false positives, with an emphasis on early recovery. In order to compare ROC curves based on these parameters, the normalized square root area under the curve (NSQ_AUC) was calculated as described previously [40] using Eq 1.

$$NSQ\_AUC=100*\frac{SQ\_AUC-SQ\_AUC_{random}}{SQ\_AUC_{perfect}-SQ\_AUC_{random}}$$ (1)

Its definition builds on the area under the curve (AUC) definition by putting emphasis on the early recovery, as well as normalizing the results between perfect recovery (NSQ_AUC = 100) and random recovery (NSQ_AUC = 0). Negative NSQ_AUC values represent ROC curves that discriminate for false positives. In order to establish statistical significance in VS performance, we have also calculated the mean NSQ_AUC ± standard error of the mean (S.E.M.) of the three docked library repeats. For more than two binding pocket comparisons, we performed a one-way ANOVA followed by a Tukey multiple comparison test (S2–S6 Tables). For comparisons between two binding pockets, we performed an unpaired t-test with Welch’s correction (S7 and S8 Tables).

An additional measure of VS performance was used to assess the known ligand enrichment within fractions of the ranked screened database (combined known ligands and decoys). In this study, enrichment factors (EFs) [41] were used to assess the early recovery of a binding pocket for each known ligand chemotype of the target receptor described above. Thus EF values were plotted for each chemotype at 2, 5 and 10% of the ranked screened database noted EF2, EF5 and EF10, respectively (S7, S8, S9, S10, S11, S12 and S13 Figs). Eq (2) describes the calculation of EF at a fraction of the ranked screened database x (i.e. 2, 5, or 10), where TP represents the total count of true positives of a specific chemotype and N, the total count of screened compounds in the database. TP_x_ represents the count of true positives of that chemotype within the fraction x of the screened database, and N_x_ represents the count of compounds within that fraction.

$$EF_{x}=\frac{TP_{x}/N_{x}}{TP/N}$$ (2)

All tasks described in the present section, namely the setup of VS experiments including preparation of target and libraries, execution on clusters, extraction of data and plotting of ROC curves with NSQ_AUC calculations as well as EF bargraphs were performed using the open source set of Python (www.python.org) scripts toolbx_vs (https://github.com/thomas-coudrat/toolbx_vs). This uses the following libraries: Matplotlib [42], Numpy [43] and scikit-learn [44].

### Binding pocket analysis

Each group of GPCR/ligand complexes were first superimposed. Root mean square deviations (RMSDs) were calculated on heavy atoms. Two RMSD comparisons were performed: ligand RMSD and binding pocket RMSD. The ligand RMSD was calculated, without further superimposition, in order to capture their relative orientation but also their relative position within the binding pocket. The binding pocket was defined as the combination of residues within 5 Å of the bound ligand in all ligand/receptor complexes compared. The binding pocket RMSD was calculated on heavy atoms of matching residues.

Docking of the cognate ligand for each binding pocket was performed using the same docking parameters as described for the VS. The score and conformation of the best scoring docked pose out of the three repeats was selected. The score of this docked pose was extracted and the RMSD to the crystal ligand of that binding pocket calculated as described above.

ICM offers an interactive scoring function that outputs a predicted score for a ligand/receptor complex in-place, with no conformational sampling. ICM scoring contains energy terms, namely solvation electrostatics and internal forcefield strain energy change, which are calculated as a difference between the ligand free and bound state. In regular scoring of a ligand/receptor complex during a docking procedure, ICM samples the ligand conformation prior to docking and the lowest energy conformation is used as the reference free state for energy calculation. As no sampling is performed in ICM’s interactive scoring, this score should not directly be compared to docking scores. In this study, ICM’s interactive scoring on X-ray ligand/receptor complexes was used in order to evaluate their predicted interaction strength. ICM’s interactive scoring was subsequently evaluated on docked ligand/receptor complexes to enable comparisons of predicted interaction strength with X-ray ligand receptor complexes. ICM’s interactive scoring is thus used as a quantitative evaluation of the ligand/receptor interaction.

IFPs describe the qualitative nature of interaction between a ligand and its binding pocket and have been identified as an effective method to post process docking poses of ligands in VS to identify those with a desired function (i.e. agonist vs antagonist)^11^. In this study, we assess IFPs of co-crystal structures and compared these to their VS performance. IFPs were computed by calculating distances and orientation between sets of atoms to define the ligand/receptor interaction pattern. The interaction types considered in this study include hydrophobic contact, hydrogen bond donor and acceptor, weak hydrogen bond donor and acceptor, ionic interaction and aromatic contact. The exact parameters used to define these interactions were described previously [45]. IFPs were generated using toolbx_pdb (https://github.com/thomas-coudrat/toolbx_pdb), a set of Python scripts for manipulation and execution of tasks on protein structure ensembles. These scripts use the libraries Matplotlib [42], Numpy [43], SciPy [46], scikit-learn [44] and the IFP implementation uses OpenEye OEChem toolkit [38] version 2014.10.2. Considering the rules and cutoffs that define the presence of an interaction using toolbx_pdb may not be identical as those defined in ICM’s forcefield, IFPs are used in this study as a tool to identify likely interactions rather than stating their absolute presence.

The resolution of X-ray crystal structures is an overall data quality metric for the model created from electron density (Table 1). Real-space correlation coefficient (RSCC) data were obtained for the binding pockets of all structures used in this study and plotted alongside B-factor values (S13, S14, S15, S16, S17, S18, S19 and S20 Figs). RSCC plots provide a residue per residue, as well as ligand, assessment of fit between the model and the electron density map that was used to generate it. A weak correlation, below a RSCC cutoff of 0.8, indicates a poor fit with electron density, which indicates either a lack of order in the modeled region, or errors in the model [47]. A problematic residue or ligand is further confirmed when low RSCC is correlated with high B-factor value. Of note, X-rays can cause radiation damage that would negatively affect the RSCC value of the damaged residue. Residues with potential for damage includes aspartic and glutamic acids, cysteines involved in disulfide bridges, methionines and tyrosines [48]. For this reason, in the current study, conclusions were not drawn based on low RSCC values associated to the aforementioned residues.

The software suite Phenix [49] was used to generate RSCC data that was plotted with in-house scripts. The Phenix utility was used to download the PDB data and convert reflection files to the MTZ format. A CIF file with the final geometry from file was downloaded for each non-protein and non-water molecule in the PDB file based on their 3-letter code, using the Phenix program eLBOW. Finally, Phenix was used to run the comprehensive validation, which computed the RSCC values that were later plotted with the scripts. R-free flags were missing from the reflection file for the following structures: 2VT4, 2Y00 and 2Y01. R-free data is not required for RSCC computation, hence the reflection files were edited with Phenix’s reflection file editor. After loading the reflection file, amplitude or intensity array was added, but R-free array was not. The R-free flags were then generated and extended to full resolution range.

The docked pose of the cognate ligand from each complex was analyzed based on its ICM docking score. Computing the RMSD on heavy atoms without superimposition facilitated comparisons between the docked pose and the X-ray pose. Additionally, the ICM interactive score was calculated for each of the docked ligand/receptor complexes.

## Results and discussion

### GPCR ligand libraries

The GLL was generated by retrieving known ligand information from the PubChem database [31], which provides 2D and 3D models of molecules. For a molecule whose activity is known to be associated with a defined enantiomeric state, both the 2D and 3D models are of the enantiomeric state. However, for molecules where activity was defined from a racemic sample, PubChem’s 2D data reflects this by not assigning the state of chiral centers, and by attributing an arbitrary enantiomeric state to the 3D molecule, which may in effect be an inactive substance. In this study, we addressed this by using the racemic GLL/GDD and sampling all enantiomeric states for each ligand that had chiral centers.

Although data analysis in this study focused on the results obtained using the racemic GLL/GDD, we did also screen the original GLL/GDD for comparison. All results were analyzed using the computed NSQ_AUC value of ROC curves from both known ligand against decoys and agonists against inhibitors VS (S2 Fig). Overall, the NSQ_AUC values obtained between racemic and original GLL/GDD were similar for all groups of binding pockets, although in many cases the racemic GLL/GDD performed slightly better than the original GLL/GDD. The naltrindole-bound (NAL) DOR binding pockets produced low NSQ_AUC scores for all VS run with both the racemic GLL/GDD and the original GLL/GDD, even after increasing the docking effort parameter from 5 to 10 (S2 and S21 Figs). This may be due in part to the complex chirality of NAL, which is shared amongst many DOR inhibitors.

### AA2AR

**Fig 1:**
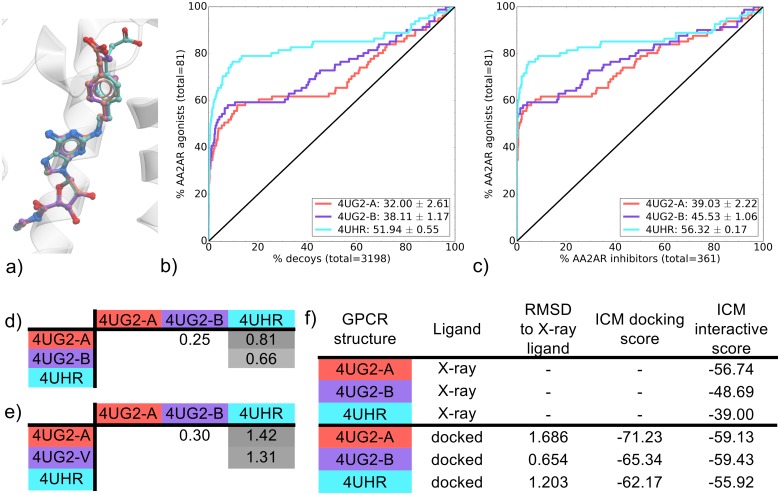
Comparison of AA2AR CGS-bound binding pockets (4UG2-A, 4UG2-B, 4UHR). (a) binding pose overlay, (b, c) binding pocket virtual screen results displayed as ROC curves of (b) AA2AR agonists against decoys and (c) AA2AR agonists against AA2AR inhibitors. The ROC curves are representations of the VS, picking the best scoring ligand after docking three independent times. A black line depicts the hypothetical random recovery of true positives. The rank of the docked co-crystal ligand relative to the percentage false positives is identified with a vertical dashed line. All vertical lines are drawn but some may not be visible as they are hidden by the main curve and/or the y-axis. The inset values are the mean NSQ_AUC ± S.E.M. of three independent experiments. Statistical significance of binding pockets is reported in S2 Table. (d, e) Heavy atom RMSD comparison of X-ray structure with (d) bound ligands and (e) binding pocket residues. (f) Comparison of X-ray structures and docked poses: RMSD to X-ray ligand, ICM docking score of the docked ligand and ICM interactive scores.

Three AA2AR CGS-bound co-crystal structures that were elucidated in the same study contain identical thermostabilizing mutations and were solved at the same resolution (2.6 Å) (Table 1). One of these thermostabilizing mutations was present in the binding pocket, but as it was present in all structures, VS performance comparison between these structures was valid, providing an even comparison between the three pockets. All three CGS-bound AA2AR binding pockets showed good recovery of known agonists against decoys, but 4UHR outperformed the others with around 80% recovery (compared to 60%) within 10% of the screened decoys (Fig 1b and S8 Table). In terms of agonist versus inhibitor recovery, the three binding pockets also performed well with again 4UHR outperforming 4UG2-A and 4UG2-B (Fig 1c). This difference in agonist enrichment was attributed mostly to 4UHR being more selective for chemotype cluster A (CGS-like) in early recovery with EF2 values of 16.8, 17.7 and 26.6 for 4UG2-a, 4UG2-b and 4UHR, respectively (S7 Fig).

**Fig 2:**
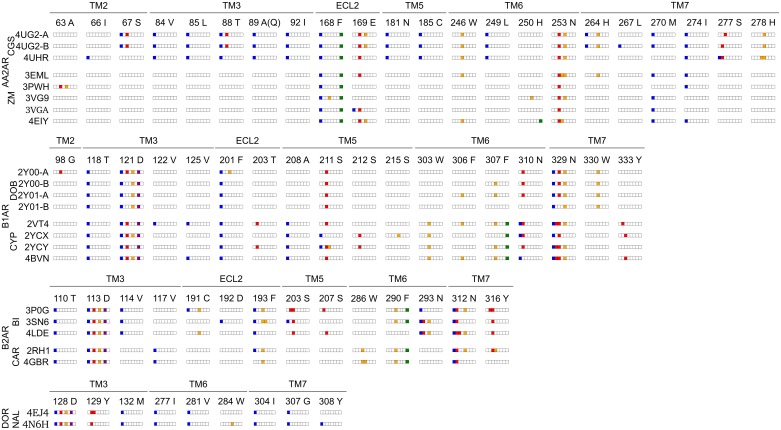
Structural interaction fingerprints for the seven groups of co-crystal X-ray structures. AA2AR CGS-bound and ZM-bound, B1AR DOB-bound and CYP-bound, B2AR BI-bound and CAR-bound, DOR NAL-bound. Interactions were determined using toolbx_pdb between the bound ligand and its receptor. Interaction types include hydrophobic (blue), hydrogen bond donor and acceptor (red), weak hydrogen-bond donor and acceptor (orange), ionic (purple) and aromatic (green). White denotes the absence of interaction. Residues forming the binding pocket are annotated by residue type, residue number and location in the 7TM domain. For AA2AR, residue 89 was mutated to A in CGS-bound binding pockets and was wild type (Q) in ZM-bound binding pockets. Co-crystal structure of the same GPCR but different co-crystal ligand (agonist/inhibitor) are aligned to highlight the differences in interaction pattern.

Visualization of CGS binding poses in 4UG2-A, 4UG2-B and 4UHR revealed a similar adenosine moiety conformation but differed slightly in the CGS interfaces with the loop region of the receptor (Fig 1a). These differences were identified with RMSD comparisons on the ligands, showing 4UHR were more distant than both 4UG2 chains (Fig 1d). The same is true for the binding pocket conformation (Fig 1e). These differences translate to small distinctions in the ligand/receptor interaction pattern in the 4UHR co-crystal relative to the 4UG2 chains (Fig 2). Despite 4UHR containing the least number of contacts between the three complexes, it bears essential contacts with the adenosine moiety of the CGS compound with ECL2 (E-168 and E-169), TM6 (L-249 and N-253) and TM7 (S-277 and H-278). Both 4UG2 A and B complexes also share these contacts, in addition to interactions specific to the CGS chemotype. As the vast majority of AA2AR agonists are based around the adenosine substructure (S1 Table), specific interaction patterns of both 4UG2 binding pockets with CGS moieties that are not part of the adenosine structure may impact on their overall recovery rate of known ligands compared to 4UHR. This outcome doesn’t preclude the use of 4UG2 X-rays structures, as indeed they have good NSQ_AUC values for both agonists vs decoys and agonists vs inhibitors. Indeed, the predicted interaction strength gives favorable scores for all three binding pockets studied. The high performance of all three CGS-bound binding pockets in recovering agonists over inhibitors may be influenced by presence of the adenosine moiety in almost all AA2AR agonists, creating a favorable conformation where adenosine can bind, thus scoring these higher than AA2AR inhibitors. Additionally, the thermostabilizing mutations present on all CGS-bound structures, may impact on AA2AR inhibitor docking. This finding for AA2AR is in agreement with a study by Kooistra and coworkers on the B2AR [11], where using the smallest subset of key IFPs to post process docking poses in rescoring, yielded the best VS outcome.

**Fig 3:**
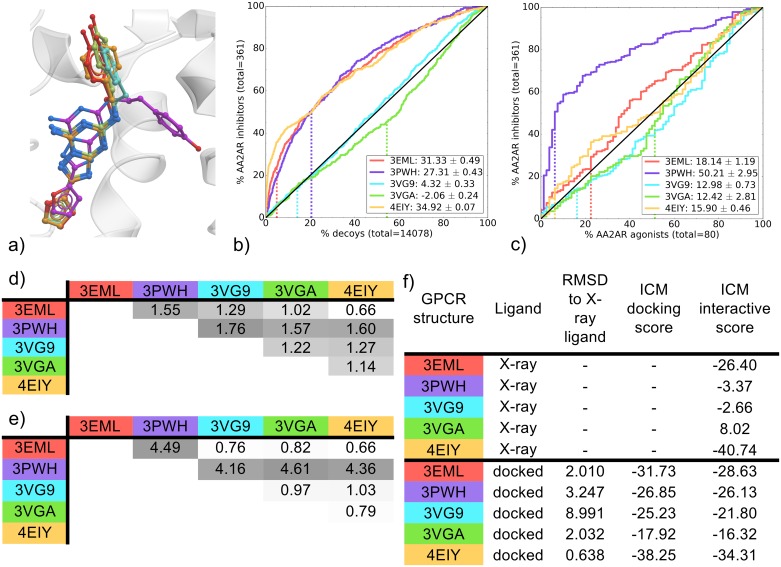
Comparison of AA2AR ZM-bound binding pockets (3EML, 3PWH, 3VG9, 3VGA and 4EIY). (a) Binding pose overlay. (b, c) Binding pocket virtual screen results displayed as ROC curves of (b) AA2AR inhibitors against decoys and (c) AA2AR inhibitors against AA2AR agonists. The ROC curves are representations of the VS, picking the best scoring ligand after docking three independent times. A black line depicts the hypothetical random recovery of true positives. The rank of the docked co-crystal ligand relative to the percentage false positives is identified with a vertical dashed line. All vertical lines are drawn but some may not be visible as they are hidden by the main curve and/or the y-axis. The inset values are the mean NSQ_AUC ± S.E.M. of three independent experiments. Statistical significance of binding pockets is reported in S3 Table. (d, e) Heavy atom RMSD comparison of X-ray structure with (d) bound ligands and (e) binding pocket residues. (f) Comparison of X-ray structures and docked poses: RMSD to X-ray ligand, ICM docking score of the docked ligand and ICM interactive scores.

The five ZM-bound binding pockets compared were from crystal structures that used combinations of mutations, insertions and antibodies to stabilize the protein for crystallography. The resolution for these structures range from 1.8 Å for 4EIY to 3.3 Å for 3PWH (Table 1). Interestingly, poor cognate docking performance was reported for the lower resolution structures (3PWH and 3VGA) in a previous study [12]. In the current study, poor cognate docking performance was unique to ZM-bound AA2AR structures (Fig 3f) a result that corroborates the findings of Ciancetta and coworkers [12]. Specifically, a high RMSD of almost 9 Å for the cognate docked pose in 3VG9 arises as the ZM docked pose was flipped 180 degrees compared to the X-ray ligand. Interestingly, the docked pose in 3PWH, with a 3.2 Å observed difference from the X-ray ligand, does not adopt a unique conformation (as observed in the crystal structure), but instead is oriented in a similar fashion to the other AA2AR ZM-bound co-crystals (S8 Fig). Moreover, the pockets clustered into two groups when comparing inhibitor recovery vs decoys: 3VG9 and 3VGA were close to random, while the remaining three binding pockets 3EML, 3PWH and 4EIY showed inhibitor recovery (Fig 3b) with above 35% of known AA2AR inhibitors recovered at 10% of decoys. However, the 4EIY binding pocket, performed significantly better than the other two (S3 Table), reaching 40% inhibitor recovery within 10% of decoys and also recovered ZM earlier in the screen relative to 3EML and 3PWH that ZM within the top 20% of decoys recovered. Comparing EF5 values for inhibitor chemotypes, this difference in performance of the top three binding pockets comes from their capacity to recover non-ZM chemotypes while the other binding pockets could not. All three had similar EF5 values for chemotype B (ZM-like), but 4EIY was more versatile at recovering alternate chemotypes A and C (S9 Fig).

Surprisingly, the binding pockets did not group in the same manner when comparing inhibitor/agonist recovery rates. Here, the 3PWH binding pocket was highly selective for AA2AR inhibitors over agonists, while the remaining binding pockets were close to random selectivity (however, 3EML and 4EIY were both better then 3VG9 and 3VGA) (Fig 3c). Interestingly, 3VG9 and 3VGA were solved using the same set of thermostabilizing mutations at two different resolutions of 2.7 Å and 3.1 Å, respectively, and exhibit a similar VS performance pattern. This resolution is close to 3EML (2.6 Å) and 3PWH (3.3 Å) that exhibit better VS performance indicating a limitation in the sole use of resolution for binding pocket selection for VS.

To summarize, the binding pockets 3EML, 3PWH and 4EIY showed the best VS performance. Within these best performers, 4EIY was the best at inhibitor recovery over decoys and 3PWH at inhibitor selectivity over agonist. Looking at the superimposed ZM-bound structures, all but one share a common conformation, with 3PWH being the exception (Fig 3a). All complexes share a common aromatic interaction with E-168 (ECL2) and a polar interaction with N-253 (TM6) (Fig 2). Analyzing similarities across complexes in more detail shows that 3EML and 4EIY are the closest in ligand conformation, while 3PWH unsurprisingly is the most distant (Fig 3d); with over 4 Å difference in RMSD with other binding pocket conformation (Fig 3e). Similarly to their high mean NSQ_AUCs in inhibitor vs decoys VS, 3EML and 4EIY also uniquely share a common set of polar contacts with E-169 (ECL2) and W-246 (TM6). 4EIY does have a better early recovery reflected in a higher mean NSQ_AUC value, due to its superior EF2 and EF5 performance for all chemotypes (S9 Fig) that could be linked to a different orientation of H-250 (TM6) that enables an additional aromatic interaction in 4EIY. Indeed, 4EIY was solved at a resolution of 1.8 Å that leads to a slightly improved fitting of side-chains, having notable impact on the VS outcome. The higher model quality of 4EIY, and to a lesser extent 3EML, can additionally be identified from the high RSCC values of their binding pocket residues (S15 Fig). In addition, interactive scoring ranks 3EML and 4EIY ligand/receptor complexes the highest with scores of -26.40 and -40.74, respectively (Fig 3f).

In this case, IFP comparisons on the starting co-crystal structures could not distinguish between high performing pockets (3EML and 4EIY) and poor pockets (3VG9 and 3VGA), but the interactive scoring betrayed a very poor fit for ZM within the 3VG9 and 3VGA binding pockets. This score might be negatively impacted by the repulsion component of the van der Waals potential as calculated by ICM^23^ that is greatly influenced by small changes in interatomic distances. This would negatively impact the predicted interaction strength between two molecules, an influence that would not be picked-up by the IFP.

The 3PWH pocket, which was significantly superior in VS of AA2AR inhibitors against agonists (Fig 3c & S5 Table), contains a different ligand binding pose compared to all the other structures, with ZM’s phenol moiety pointing towards TM2 interacting with the carboxyl group of A63 (Fig 3a). The 3PWH complex does not have all the polar contacts present in 3EML and 4EIY, and the RSCC plot of 3PWH indicates a poor fit of the ligand ZM with its electron density (S14b Fig). Despite this, the ligand’s unique orientation and its effect on the binding pocket conformation seems to favor AA2AR inhibitors over agonists. Although cognate docking in the 3PWH binding pocket does not replicate the same unique X-ray binding pose (S8 Fig), the data suggest that screening for known inhibitors benefit from the unique binding pocket conformation stabilized by ZM, whereas agonists do not.

### B1AR

**Fig 4:**
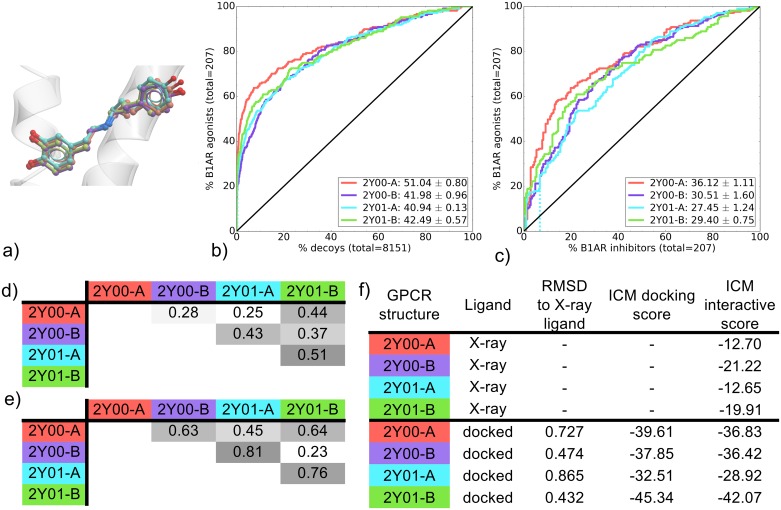
Comparison of B1AR DOB-bound binding pockets (2Y00-A, 2Y00-B, 2Y01-A and 2Y01-B). (a) Binding pose overlay. (b, c) Binding pocket virtual screen results displayed as ROC curves of (b) B1AR agonists against decoys and (c) B1AR agonists against B1AR inhibitors. The ROC curves are representations of the VS, picking the best scoring ligand after docking three independent times. A black line depicts the hypothetical random recovery of true positives. The rank of the docked co-crystal ligand relative to the percentage false positives is identified with a vertical dashed line. All vertical lines are drawn but some may not be visible as they are hidden by the main curve and/or the y-axis. The inset values are the mean NSQ_AUC ± S.E.M. of three independent experiments. Statistical significance of binding pockets is reported in S4 Table. (d, e) Heavy atom RMSD comparison of X-ray structure with (d) bound ligands and (e) binding pocket residues. (f) Comparison of X-ray structures and docked poses: RMSD to X-ray ligand, ICM docking score of the docked ligand and ICM interactive scores.

The four dobutamine-bound (DOB) B1AR binding pockets extracted from two crystal structures, showed good early recovery of known inhibitors over decoys as well as selectivity of known inhibitors over agonists. The 2Y00-A binding pocket however had the best overall VS performance, showing significantly superior mean NSQ_AUC values for both agonists over decoys and agonists over inhibitors, compared to the other DOB-bound binding pockets (Fig 4b and 4c and S4 Table). The early recovery for 2Y00-A may be influenced in part by superior EF2, EF5 and EF10 values for chemotypes A (S10 Fig), which is over represented amongst B1AR agonists (S1 Table). The similar VS performance of B1AR DOB-bound binding pockets could be expected from a set of X-ray structures obtained from the same study where slightly different crystallization conditions were used.

Most complexes exhibit polar interactions in TM3 (D-121), TM5 (S-211) and TM7 (N-310, N-329, W-330). They also all share the ionic interaction with D-121 in TM3 that is conserved amongst aminergic GPCRs. However, 2Y00-A has a different interaction pattern with additional contacts to TM2 (G-98) and ECL2 (F-201) that may be responsible for its slightly better recovery of agonists over both decoys and inhibitors, relative to the other structures.

**Fig 5:**
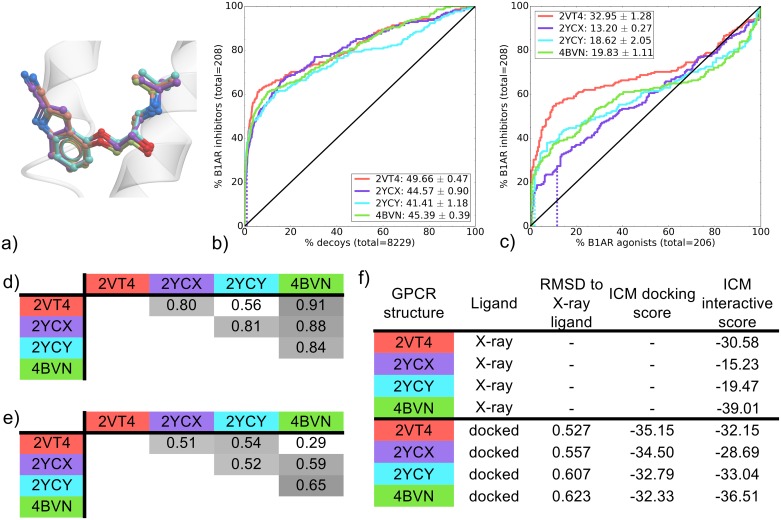
Comparison of B1AR CYP-bound binding pockets (2VT4, 2YCX, 2YCY and 4BVN). (a) Binding pose overlay. (b, c) Binding pocket virtual screen results displayed as ROC curves of (b) B1AR inhibitors against decoys and (c) B1AR inhibitors against B1AR agonists. The ROC curves are representations of the VS, picking the best scoring ligand after docking three independent times. A black line depicts the hypothetical random recovery of true positives. The rank of the docked co-crystal ligand relative to the percentage false positives is identified with a vertical dashed line. All vertical lines are drawn but some may not be visible as they are hidden by the main curve and/or the y-axis. The inset values are the mean NSQ_AUC ± S.E.M. of three independent experiments. Statistical significance of binding pockets is reported in S5 Table. (d, e) Heavy atom RMSD comparison of X-ray structure with (d) bound ligands and (e) binding pocket residues. (f) Comparison of X-ray structures and docked poses: RMSD to X-ray ligand, ICM docking score of the docked ligand and ICM interactive scores.

Cyanopindolol (CYP) was co-crystallized in four different B1AR binding pockets. All CYP-bound binding pockets performed similarly in recovery of known ligands vs decoys, as seen by mean NSQ_AUC values ranging from 41.49 to 49.66 (Fig 5b). In this case, statistical analysis points to 2VT4 as the highest performer (S5a Table). All CYP-bound binding pockets also showed selectivity for B1AR inhibitors over agonists and 2VT4 was again found to significantly outperform other binding pockets (Fig 5c and S5b Table). In both inhibitors vs. decoys and inhibitors vs. agonists, 4BVN was ranked second in mean NSQ_AUC value (Fig 5b and 5c).

Despite different resolutions (ranging from 2.10 Å to 3.25 Å), all B1AR CYP-bound structures share the same set of thermostabilizing mutations. All complexes have a common ligand/receptor ionic and polar interaction with D-121, an aromatic interaction with F-307 and a polar interaction with N-329, beyond these, each complex contains a different combination of hydrophobic and polar contacts (Fig 2). When looking at the interactive scoring, the most favorable scores are predicted for 2VT4 and 4BVN with -30.58 and -39.01, respectively (Fig 5f). To summarize, 2VT4 showed a significantly superior VS performance with 4BVN also performing well in both retrospective screens. While an interaction pattern responsible for this result is difficult to identify, the interactive score does pick out the best binding pockets, which incidentally are the closest in conformation (Fig 5e).

### B2AR

**Fig 6:**
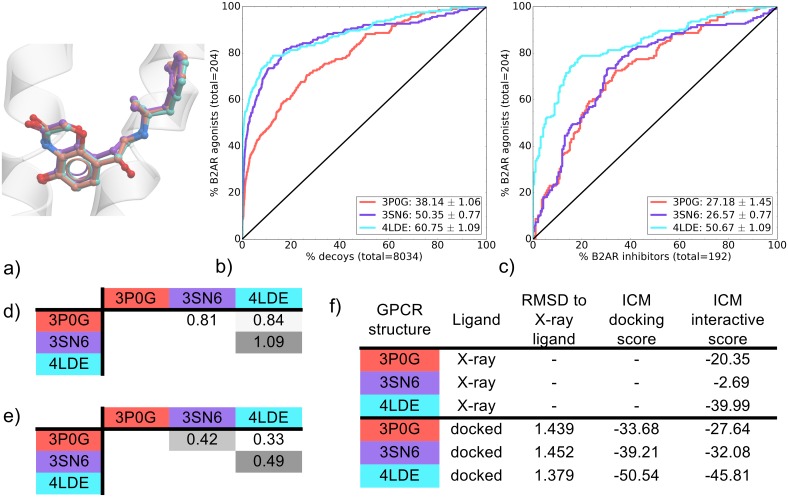
Comparison of B2AR BI-bound binding pockets (3P0G, 3SN6 and 4LDE). (a) Binding pose overlay. (b, c) Binding pocket virtual screen results displayed as ROC curves of (b) B2AR agonists against decoys and (c) B2AR agonists against B2AR inhibitors. The ROC curves are representations of the VS, picking the best scoring ligand after docking three independent times. A black line depicts the hypothetical random recovery of true positives. The rank of the docked co-crystal ligand relative to the percentage false positives is identified with a vertical dashed line. All vertical lines are drawn but some may not be visible as they are hidden by the main curve and/or the y-axis. The inset values are the mean NSQ_AUC ± S.E.M. of three independent experiments. Statistical significance of binding pockets is reported in S6 Table, (d, e) Heavy atom RMSD comparison of X-ray structure with (d) bound ligands and (e) binding pocket residues. (f) Comparison of X-ray structures and docked poses: RMSD to X-ray ligand, ICM docking score of the docked ligand and ICM interactive scores.

The BI-bound B2AR pockets were stabilized differently for crystallization using combinations of insertions, mutations and stabilizing molecules. The latest structure to be solved, 4LDE, has the best resolution at 2.79 Å and also has very high RSCC values for its ligand and binding pocket residues (S18c Fig). The three binding pockets compared in this study had very different VS performance profiles. All three were able to distinguish known B2AR agonists from decoys with mean NSQ_AUC values ranging from 38.14 ± 1.06 (3P0G) to 60.75 ± 1.09 (4LDE). 4LDE and 3SN6 were particularly effective with their representative ROC curve showing a recovery of known agonists very early on, reaching around 70% and 60% recovery, respectively, of known agonists within 10% of decoys (Fig 6b). This early enrichment was associated with a superior early recovery of known ligands of chemotype A (S12 Fig), a different chemotype than that of the co-crystallized ligand (BI-167107) (BI) (S1 Table). When the three binding pockets were compared for their ability to distinguish B2AR agonists from inhibitors, the 4LDE binding pocket was significantly better than the other two with a mean NSQ_AUC value of 50.67 ± 1.09 (Fig 6c and S6 Table). In this case, the best VS performer corresponds to the best X-ray structure resolution. Additionally, this showcases another example where the best binding pocket for recovering known ligands against decoys also performs the best for agonist/inhibitor distinction.

Comparing the ligand/receptor interaction patterns, all three complexes share a common polar interaction network in TM3 (D-113), TM5 (S-203) and TM7 (N-312, Y-316) as well as the conserved ionic interaction D-113, for aminergic GPCRs (Fig 2). However, the top two VS performers 4LDE and 3SN6 are differentiated from 3P0G by the presence of three key interactions in both structures of BI with TM6 and TM7; polar interactions with N-293 (TM6) and for 4LDE, a double hydrogen bond interaction with N-312 (TM7). Combined, these additional contacts may contribute to increased recognition of known agonists over decoys and/or inhibitors. Interestingly, 4LDE is the only binding pocket without a ligand contact to F-290 in TM6. This provides additional space in the binding pocket that may be critical in allowing the larger agonists of chemotype A (S1 Table) to be greatly enriched at EF2 in 4LDE (S11 Fig).

Comparing the ICM interactive scores for each binding pocket, 4LDE is favored at -39.99, with 3SN6 and 3P0G both scored too low to be compared to one another (-2.69 and -20.35, respectively) (Fig 6f). The low RSCC value of the ligand BI in the binding pocket of 3SN6 may contribute to its very low score (S18b Fig). It should also be noted that missing side-chains were added to the binding pocket of 3SN6 followed by optimization prior to VS. Although the same optimization procedure was applied on all three BI-bound binding pockets, the addition of these key side-chains in ECL2 (F193, F194, T195) by the ICM software using its forcefield and scoring function may have contributed to improving the VS performance of 3SN6.

**Fig 7:**
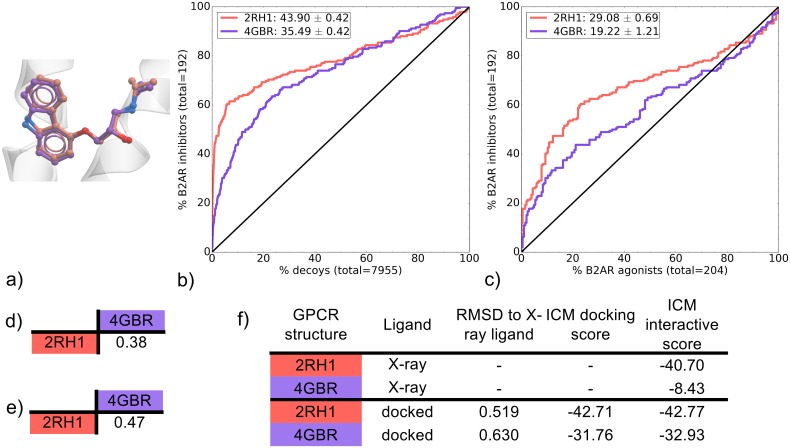
Comparison of B2AR CAR-bound binding pockets (2RH1 and 4GBR). (a) Binding pose overlay. (b, c) Binding pocket virtual screen results displayed as ROC curves of (b) B2AR inhibitors against decoys and (c) B2AR inhibitors against B2AR agonists. The ROC curves are representations of the VS, picking the best scoring ligand after docking three independent times. A black line depicts the hypothetical random recovery of true positives. The rank of the docked co-crystal ligand relative to the percentage false positives is identified with a vertical dashed line. All vertical lines are drawn but some may not be visible as they are hidden by the main curve and/or the y-axis. The inset values are the mean NSQ_AUC ± S.E.M. of three independent experiments. Statistical significance of binding pockets is reported in S7 Table. (d, e) Heavy atom RMSD comparison of X-ray structure with (d) bound ligands and (e) binding pocket residues. (f) Comparison of X-ray structures and docked poses: RMSD to X-ray ligand, ICM docking score of the docked ligand and ICM interactive scores.

The binding pockets of 2RH1 and 4GBR are both carazolol-bound (CAR) B2AR X-ray structures that were solved with a resolution of 2.4 Å and 3.99 Å, respectively (Table 1). In both cases T4L insertion and thermostabilizing mutations were used to facilitate crystal formation; ICL3 T4L insertion and one mutation for 2RH1, N-term T4L insertion and three mutations for 4GBR. Although both binding pockets recovered inhibitors from decoys, 2RH1 significantly outperformed 4GBR (Fig 7b and S7 Table). This can be attributed to its versatility in identifying ligands of multiple chemotypes as it achieved superior EF2, EF5 and EF10 values for all three major chemotypes A, B (CAR-like) and C (S13 Fig). Both binding pockets were also compared for their recovery of B2AR inhibitors over agonists, where 2RH1 also outperformed 4GBR in overall mean NSQ_AUC score (Fig 7c).

Both CAR-bound B2AR X-ray structures are similar in binding pocket and bound ligand conformation (Fig 7d and 7e). This observation extends to the ligand/receptor interactions, as both complexes share a very similar pattern. One key difference is a set of polar contacts between ligand and Y-316 (TM7) only found in 2RH1 (Fig 2). The precise orientation of Y-316 in this structure provides an additional anchor point that may contribute to an improved VS performance for 2RH1. As has been the case for previous groups of binding pockets, the interactive score correlates with VS performance with 2RH1 scoring -40.70 compared to a poor score of -8.43 for 4GBR (Fig 7f).

### DOR

**Fig 8:**
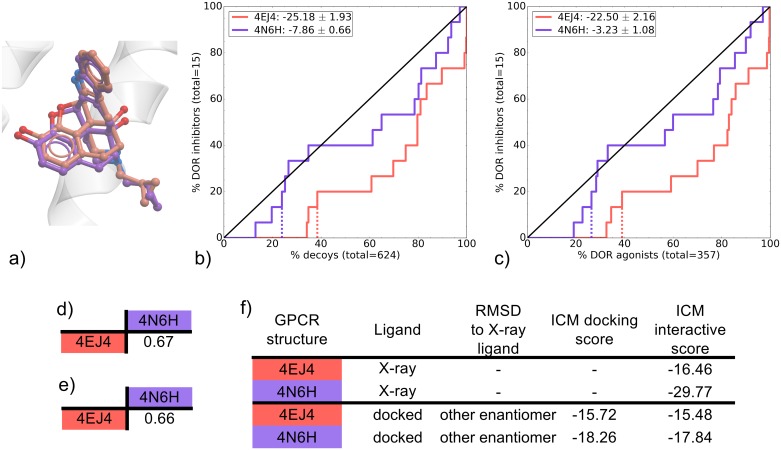
Comparison of DOR NAL-bound binding pockets (4EJ4 and 4N6H). (a) Binding pose overlay. (b, c) binding pocket virtual screens results displayed as ROC curves of (b) DOR inhibitors against decoys and (c) DOR inhibitors against DOR agonists. The ROC curves are representations of the VS, picking the best scoring ligand after docking three independent times. A black line depicts the hypothetical random recovery of true positives. The rank of the docked co-crystal ligand relative to the percentage false positives is identified with a vertical dashed line. All vertical lines are drawn but some may not be visible as they are hidden by the main curve and/or the y-axis. The inset values are the mean NSQ_AUC ± S.E.M. of three independent experiments. Statistical significance of binding pockets is reported in S8 Table. (d, e) Heavy atom RMSD comparison of X-ray structure with (d) bound ligands and (e) binding pocket residues. (f) Comparison of X-ray structures and docked poses: RMSD to X-ray ligand, ICM docking score of the docked ligand and ICM interactive scores.

Two DOR naltrindole-bound (NAL) binding pockets were compared. 4EJ4 is the *mouse* receptor whereas 4N6H is a *human* receptor, however there are only three amino acids that differ between the two species, all of which are located away from the binding pocket (Table 1). Known ligands for the *human* DOR were screened in this study on both binding pockets. The VS performance was poor for both DOR binding pockets (Fig 8b and 8c). While ROC curves for 4N6H were close to random in both inhibitors vs decoys and inhibitors vs agonists, they were significantly better than 4EJ4, which identified false positives at a higher rate than true positives (S8 Table). Only 15 known DOR inhibitors were screened in the DOR VS, and indeed none of these molecules were recovered at EF10 (data not shown). Increasing the docking effort parameter from 5 to 10 did not greatly improve these results (S21 Fig). The NSQ_AUC values were slightly better when screened with the original GLL/GDD in comparison with the racemic GLL/GDD, but these still remained very poor (S2 Fig). These results make it difficult to draw conclusions on the overall VS performance of these binding pockets. Additionally, while waters are not taken into account in this study’s docking procedure, their inclusion may positively affect the VS outcomes. Indeed it was shown previously that a κ-opioid receptor (KOR) model including key crystal waters displayed superior performance in VS [50]. Nevertheless, some relevant information can be drawn from these results. The two co-crystal ligand/receptor interaction patterns are very similar, with NAL forming hydrogen bonds with TM3’s D-128 and Y-129 as well as an ionic interaction with D-128 in both complexes (Fig 2). However, the interactive scoring differs with a value of -16.46 for 4EJ4 compared to a much more favorable -29.77 for 4N6H (Fig 8f). This corresponds to the resolution of the respective structures, where 4EJ4 was solved at 3.4 Å and 4N6H was solved at a very high resolution of 1.8 Å. As in other examples, the best performing binding pocket corresponds to the one with the highest resolution as well as the highest ICM interactive scoring for the X-ray ligand.

## Conclusions

This study and others highlight that GPCR structure(s) used at the start of a prospective VS will dictate the success of a SBDD program. We took advantage of the increasing number of GPCR X-ray crystal structures available to identify key criteria that may contribute to VS performance. By selecting a range of GPCR complexes bound to both agonists and inhibitors, we aimed to draw conclusions that are broadly applicable and may aid in selection of a structure for VS programs where multiple are available. VS performance of each GPCR binding pocket was evaluated by comparing recovery rates of known ligands against decoys, as well as agonists against inhibitors. While our study was performed with the use of a single docking software (icm), similar results in VS selectivity were observed by Kooistra and coworkers for the B1AR and B2AR [11] using a different docking software, highlighting that our findings will likely extend to the use of other docking software. The case study on the B1AR and B2AR identified that IFPs used to post process docking poses enhanced the retrieval of ligands in VS with select pharmacology (i.e. inhibitors vs agonists or vice versa). However, this method requires extensive knowledge of the desired IFP and, just as in the case of VS in the absence of IFP rescoring, the crystal structure used in VS also affected the degree to which the selected IFP was able to influence the VS enrichment [11]. In this current study, the focus was on identification of criteria to guide the selection of a co-crystal structure template for use in VS when more than one is available to enhance the chances of a successful outcome.

In addition to the B1AR and B2AR, two additional class A GPCRs (AA2AR and DOR) were also investigated and larger libraries of known ligands and decoys were used for retrospective screens compared to those employed in the study by Kooistra and coworkers [11]. This enabled an in depth analysis of the VS outcomes that were visualized using ROC curve representations, where relative rank of ligands was based on their best out of three docking scores. Additionally, these three repeats allowed to calculate mean NSQ_AUC ± S.E.M., which places emphasis on early recovery, and enables comparison of VS performance significance amongst binding pockets. Additionally, screening exhaustive known ligand libraries enabled assessment of the recovery of different known ligand chemotypes using EF barcharts. As numerous known ligands may be attributed the wrong enantiomeric state in the original GLL/GDD, the racemic GLL/GDD that generally had improved performance in VS were used.

**Fig 9:**
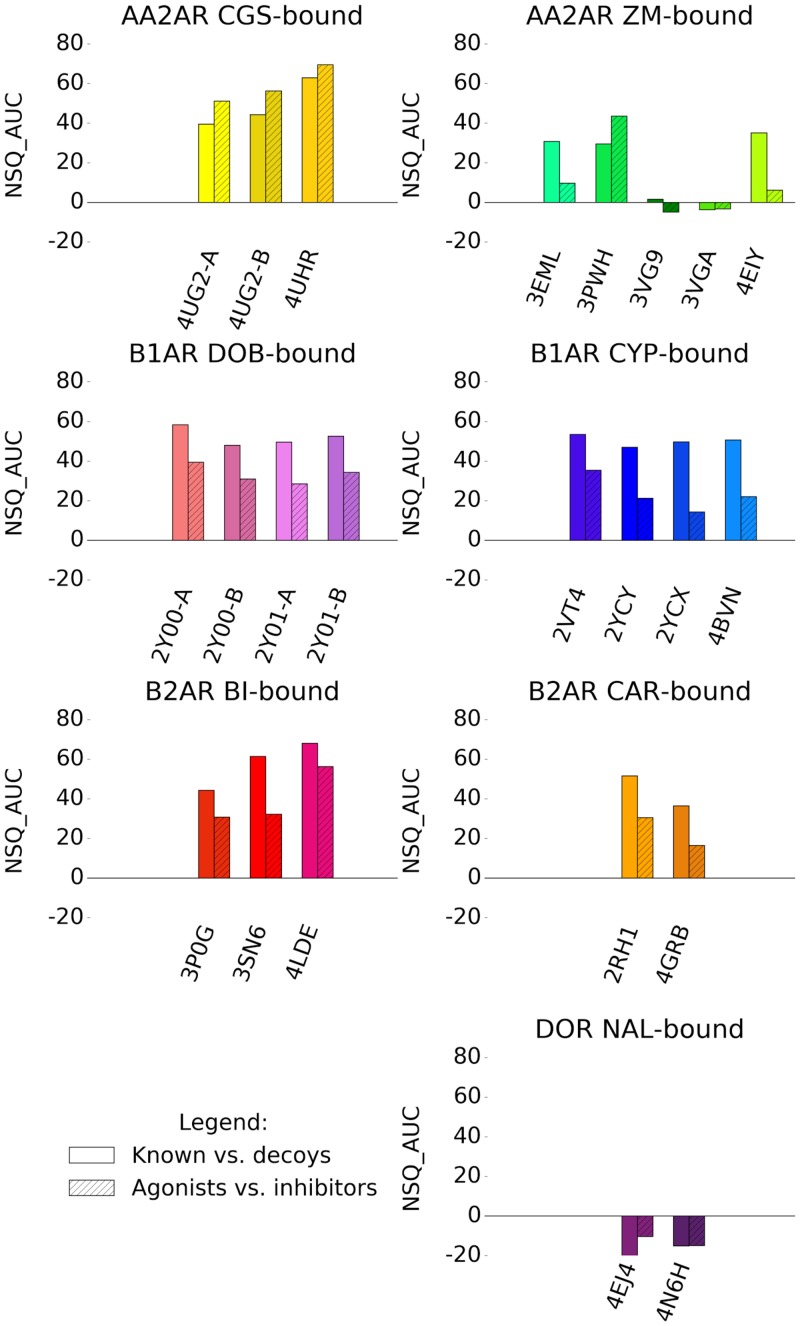
Comparison of VS performance on groups of binding pockets bound by the same ligand. Compared are recovery of known ligands against decoys shown in plain, and recovery of agonists against inhibitors (or vice-versa) shown in hatched. NSQ_AUCs values emphasize early recovery of a ROC curve. These are normalized between perfect (NSQ_AUC = 100) and random (NSQ_AUC = 0) recovery. A negative NSQ_AUC value indicates a ROC curve below random.

Interestingly, we observed that oligomers identified within the same X-ray structure can perform differently in VS, however a greater difference in both performance and chemotype selectivity profile was seen in binding pockets obtained from different X-ray structures. Whereas a retrospective VS on known ligands and decoys across numerous binding pockets can be computationally costly, in the majority of cases, VS performance for agonists vs inhibitors followed the same trend exhibited by known ligands against decoys recovery (Fig 9). The selectivity task has the additional benefit of screening for the required pharmacology representing a thorough test on the viability of a binding pocket for prospective VS, while being much less computationally expensive compared with vs decoys. Known ligand libraries tested represented various different chemotypes for each GPCR target. In the current study the best VS performance was achieved from binding pockets that were versatile in recovering a wide range of known ligand chemotype.

GPCR X-ray structures are obtained through the use of a number of experimental and protein engineering methods that include thermostabilizing mutations, insertion of proteins within regions of the GPCR, deletion of flexible regions and stabilization by other molecules such as nanobodies. These modifications facilitate the crystallization of the receptors by stabilizing them in a single conformational state and enhancing crystal contacts. The current study does not point to a particular set of crystallization methods that correlate with their VS performance. The current de-facto metric used to evaluate an X-ray structure for VS, is its resolution and indeed, in most cases, a higher resolution was indicative of a better VS performance, presumably because a higher resolution enables a better fit of side-chains and ligand into the electron density, therefore producing a better model. This was complemented in some cases by the analysis of RSCC plots, which inform more specifically on the quality of the modeled binding pocket and bound ligand. Interestingly, the differential performance of binding pockets in VS was always dictated by extremely small differences in the relative position of atoms of the screened binding pocket model. Cases where lower resolution structures showed better VS performance than others of higher resolution prompts the need for additional metrics that may aid in identifying the best binding pocket to select for SBDD.

In this study, we undertook a quantitative analysis of all ligand/receptor interactions using the ICM scoring function used in the VS experiments. The ICM interactive scoring provides a value of the scoring function’s interpreted interaction strength between ligand and receptor. We identified ICM interactive scores as an indicator of binding pocket VS performance. This is particularly true for ICM scores of -20 or better, as lower scores tend to be less meaningful. An additional tool complementing the ICM interactive score analysis was a detailed qualitative analysis of interaction patterns between the co-crystalized ligands and receptors. With these IFPs, we identified that the presence of specific hydrogen bond interactions in the starting structures was key to VS performance, at least for these class A GPCRs assessed, these interactions were present mostly on TM3, TM6, TM7 and for some GPCRs ECL2. Two interesting findings arise from the IFP analysis that was not captured by ICM interactive scoring. Firstly, an alternative binding pose, such as the one seen in AA2AR ZM-bound binding pockets can offer an effective binding pocket in VS, even if its ligand/receptor interaction is not favorably scored by ICM. Secondly, as seen in AA2AR CGS-bound binding pockets, an increased number of polar contacts between ligand and receptor may hinder the recovery of known ligands outside the chemotype of the ligand used for crystallization, as that ligand/receptor interaction is too specific to the chemotype of the co-crystallized ligand. Although this does not preclude the use of such a binding pocket for prospective VS, it is information that one should keep in mind in a SBDD program, especially if the goal is to identify new scaffolds. In cases such as the B2AR BI-bound binding pockets (where all three binding pockets performed well in VS) the presence of additional polar and aromatic interactions in two of the structures relative to the best performer (4LDE) also hindered recovery of known ligands outside the chemotype of the co-crystallized ligand.

In a SBDD program, a prospective VS should include a step of thorough energy minimization on the ligand/receptor complex. Improving the model geometry using the same forcefield and scoring function as the ICM interactive score and docking would improve values of both. Additionally, a careful selection of binding pocket water molecules has been shown to have a positive influence on docking known ligands [50] and improving VS outcome [51]. Neither of these important factors were included in the current study, however, the use of tools that could include waters not observed in crystal structures due to low resolution may also aid in VS performance on crystal structures. To summarize, we have compared the VS performance of X-ray structures of the same GPCR bound to the same ligand and identified indications that small variations in structural features are responsible for their success in VS (Table 2). These results provide a framework to continue the development of computational tools aimed at the refinement of GPCR binding pocket conformation to improve their predictive power in VS.

**Table 2:** GPCR X-ray structure features indicative of their relative VS performance.

GPCR	Co-crystal ligand	Resolution	Interaction strength	Interaction pattern	Ligand conformation	Binding pocket conformation
AA2AR	CGS	-	-	√	√	√
AA2AR	ZM	-	√	√	√	√
B1AR	DOB	-	-	-	-	-
B1AR	CYP	-	√	-	-	√
B2AR	BI	√	√	√	-	√
B2AR	CAR	√	√	√	-	-
DOR	NAL	√	√	√	-	-

Interaction strength refers to the calculated ICM interactive scoring. Interaction pattern refers to the qualitative IFP scoring. Ligand and binding pocket conformation refer RMSD difference. No difference identified in the VS performance and features assessed between the different x-ray structures are denoted by “-” and an indication of an important feature for VS performance is denoted by “√”.

## Supporting information

S1 Fig Comparison of VS performance with and without hydrogen bond optimization. The known ligands vs. decoys and agonist vs. inhibitors (or vice versa) are compared respectively for (a,b) AA2AR-ZM 3EML, (c,d) AA2AR-ZM 4EIY, (e,f) B1AR-DOB 2Y00-A, (g,h) B1AR-CYP 2VT4, (i,j) B1AR-CYP 2YCX, (k,l) B1AR-CYP 2YCY, (m,n) B1AR-CYP 4BVN, (o,p) B2AR-BI 3P0G, (q,r) B2AR-BI 4LDE, (s,t) B2AR-BI 3SN6, (u,v) B2AR-CAR 2RH1 and (w,x) DOR-NAL 4N6H.
(PDF)

S2 Fig Ligand library original GLL/GDD vs racemic GLL/GDD NSQ_AUC comparisons. Comparison of VS performance on groups of binding pockets bound by the same ligand depending on the ligand library used for screening: original GLL/GDD (hatched) or racemic GLL/GDD (plain). Values are NSQ_AUCs calculated on ROC curves comparing a) known ligands against decoys, and b) agonists against inhibitors (or vice-versa).
(PDF)

S3 Fig Dendrogram of the ligand chemotypes for AA2AR. Known a) agonists and b) inhibitors. The number of ligands within each branch is noted. Clusters used in VS are circled and named, and the co-crystal X-ray ligand’s position is identified. The asterisk denotes the location of the chemotype cluster center for its respective chemotype cluster, no cluster center is provided for the cluster ‘other’.
(PDF)

S4 Fig Dendrogram of the ligand chemotypes for B1AR. Known a) agonists and b) inhibitors. The number of ligands within each branch is noted. Clusters used in VS are circled and named, and the co-crystal X-ray ligand’s position is identified. The asterisk denotes the location of the chemotype cluster center for its respective chemotype cluster, no cluster center is provided for the cluster ‘other’.
(PDF)

S5 Fig Dendrogram of the ligand chemotypes for B2AR. Known a) agonists and b) inhibitors. The number of ligands within each branch is noted. Clusters used in VS are circled and named, and the co-crystal X-ray ligand’s position is identified. The asterisk denotes the location of the chemotype cluster center for its respective chemotype cluster, no cluster center is provided for the cluster ‘other’.
(PDF)

S6 Fig Dendrogram of the ligand chemotypes for DOR. The number of ligands within each branch is noted. Clusters used in VS are circled and named, and the co-crystal X-ray ligand’s position is identified. The asterisk denotes the location of the chemotype cluster center for its respective chemotype cluster, no cluster center is provided for the cluster ‘other’.
(PDF)

S7 Fig Enrichment factors of AA2AR known agonist chemotypes for CGS-bound AA2AR binding pockets (4UG2-A, 4UG2-B, 4UHR). Enrichment factors at EF2, EF5 and EF10 using the 2D racemic ligand library.
(PDF)

S8 Fig Cognate docking of the ZM ligand on all AA2AR ZM-bound binding pockets (3EML, 3PWH, 3VG9, 3VGA and 4EIY). All complexes were superimposed and only one representative receptor is displayed in grey, with TM6 and TM7 omitted for clarity. Carbon atoms of the ligands are colored as follows: 3EML (red), 3PWH (purple), 3VG9 (cyan), 3VGA (green), 4EIY (yellow).
(PDF)

S9 Fig Enrichment factors of AA2AR known inhibitor chemotypes for ZM-bound AA2AR binding pockets (3EML, 3PWH, 3VG9, 3VGA, 4EIY). Enrichment factors at EF2, EF5 and EF10.
(PDF)

S10 Fig Enrichment factors of B1AR known agonist chemotypes for DOB-bound B1AR binding pockets (2Y00-A, 2Y00-B, 2Y01-A, 2Y01-B). Enrichment factors at EF2, EF5 and EF10.
(PDF)

S11 Fig Enrichment factors of B1AR known inhibitor chemotypes for CYP-bound B1AR binding pockets (2VT4, 2YCX, 2YCY, 4BVN). Enrichment factors at EF2, EF5 and EF10.
(PDF)

S12 Fig Enrichment factors of B2AR known agonist chemotypes for BI-bound B2AR binding pockets (3P0G, 3SN6, 4LDE). Enrichment factors at EF2, EF5 and EF10.
(PDF)

S13 Fig Enrichment factors of B2AR known inhibitor chemotypes for CAR-bound B2AR binding pockets (2RH1, 4GBR). Enrichment factors at EF2, EF5 and EF10.
(PDF)

S14 Fig RSCC and B-factor plots for AA2AR CGS-bound binding pockets. Assessment of local model quality for: a) 4UG2-A, b) 4UG2-B and c) 4UHR. Real-space correlation coefficient (green) and B-factor values (red) are shown for all residues of the binding pocket and the bound ligand CGS. A green dotted line cutoff value of 0.8 highlights low RSCC values.
(PDF)

S15 Fig RSCC and B-factor plots for AA2AR ZM-bound binding pockets. Assessment of local model quality for: a) 3EML, b) 3PWH, c) 3VG9, d) 3VGA and e) 4EIY. Real-space correlation coefficient (green) and B-factor values (red) are shown for all residues of the binding pocket and the bound ligand ZM. A green dotted line cutoff value of 0.8 highlights low RSCC values.
(PDF)

S16 Fig RSCC and B-factor plots for B1AR DOB-bound binding pockets. Assessment of local model quality for: a) 2Y00-A, b) 2Y00-B, c) 2Y01-A and d) 2Y01-B. Real-space correlation coefficient (green) and B-factor values (red) are shown for all residues of the binding pocket and the bound ligand DOB. A green dotted line cutoff value of 0.8 highlights low RSCC values.
(PDF)

S17 Fig RSCC and B-factor plots for B1AR CYP-bound binding pockets. Assessment of local model quality for: a) 2VT4, b) 2YCX, c) 2YCY and d) 4BVN. Real-space correlation coefficient (green) and B-factor values (red) are shown for all residues of the binding pocket and the bound ligand CYP. A green dotted line cutoff value of 0.8 highlights low RSCC values.
(PDF)

S18 Fig RSCC and B-factor plots for B2AR BI-bound binding pockets. Assessment of local model quality for: a) 3P0G, b) 3SN6 and c) 4LDE. Real-space correlation coefficient (green) and B-factor values (red) are shown for all residues of the binding pocket and the bound ligand BI. A green dotted line cutoff value of 0.8 highlights low RSCC values.
(PDF)

S19 Fig RSCC and B-factor plots for B2AR CAR-bound binding pockets. Assessment of local model quality for: a) 2RH1 and b) 4GBR. Real-space correlation coefficient (green) and B-factor values (red) are shown for all residues of the binding pocket and the bound ligand CAR. A green dotted line cutoff value of 0.8 highlights low RSCC values.
(PDF)

S20 Fig RSCC and B-factor plots for DOR NAL-bound binding pockets. Assessment of local model quality for: a) 4EJ4 and b) 4N6H. Real-space correlation coefficient (green) and B-factor values (red) are shown for all residues of the binding pocket and the bound ligand NAL. A green dotted line cutoff value of 0.8 highlights low RSCC values.
(PDF)

S21 Fig Analyzing the influence of the docking effort parameter on VS performance for DOR binding pockets (4EJ4 and 4N6H). VS results displayed as ROC curves of (a) DOR inhibitors against decoys and (b) DOR inhibitors against DOR agonists. The ROC curves are representations of the VS, picking the best scoring ligand after docking three independent times. A black line depicts the hypothetical random recovery of true positives. The rank of the docked co-crystal ligand relative to the percentage false positives is identified with a vertical dashed line. The inset values are NSQ_AUCs calculated on these representative curves.
(PDF)

S1 Table Known ligand library and decoys for each GPCR. Details about known ligand count, chiral center composition, as well as chemotype clusters names and counts. A chemical structure for the center of each chemotype cluster is represented, as well as each ligand from co-crystal X-ray structures.
(PDF)

S2 Table Statistical significance of VS performance between AA2AR CGS-bound binding pockets. One-way ANOVA was performed on mean NSQ_AUC ± S.E.M. for each of the docking experiments, followed by a Tukey multiple comparison test for a) AA2AR agonists vs. decoys (Fig 1b) and b) AA2AR agonists vs. AA2AR inhibitors (Fig 1c). A one-way ANOVA was carried out, followed by a Tukey’s multiple comparison test. Binding pocket performance was tested with P value noted as follows. *: P ≤ 0.05, **: P ≤ 0.01, ***: P ≤ 0.001, ****: P ≤ 0.0001, ns: not significantly different. Black asterisks signify the row structure is significantly better than the column structure, and vice-versa for red asterisks.
(PDF)

S3 Table Statistical significance of VS performance between AA2AR ZM-bound binding pockets. One-way ANOVA was performed on mean NSQ_AUC ± S.E.M. for each of the docking experiments, followed by a Tukey multiple comparison test for a) AA2AR inhibitors vs. decoys (Fig 3b) and b) AA2AR inhibitors vs. AA2AR agonists (Fig 3c). A one-way ANOVA was carried out, followed by Tukey’s multiple comparison test. Binding pocket performance is tested with P value noted as follows. *: P ≤ 0.05, **: P ≤ 0.01, ***: P ≤ 0.001, ****: P ≤ 0.0001, ns: not significantly different. Black asterisks signify the row structure is significantly better than the column structure, and vice-versa for red asterisks.
(PDF)

S4 Table Statistical significance of VS performance between B1AR DOB-bound binding pockets. One-way ANOVA was performed on mean NSQ_AUC ± S.E.M. for each of the docking experiments, followed by a Tukey multiple comparison test for a) B1AR agonists vs. decoys (Fig 4b) and b) B1AR agonists vs. B1AR inhibitors (Fig 4c). A one-way ANOVA was carried out, followed by Tukey’s multiple comparison test. Binding pocket performance is tested with P value noted as follows. *: P ≤ 0.05, **: P ≤ 0.01, ***: P ≤ 0.001, ****: P ≤ 0.0001, ns: not significantly different. Black asterisks signify the row structure is significantly better than the column structure, and vice-versa for red asterisks.
(PDF)

S5 Table Statistical significance of VS performance between B1AR CYP-bound binding pockets. One-way ANOVA was performed on mean NSQ_AUC ± S.E.M. for each of the docking experiments, followed by a Tukey multiple comparison test for a) B1AR inhibitors vs. decoys (Fig 5b) and b) B1AR inhibitors vs. B1AR agonists (Fig 5c). A one-way ANOVA was carried out, followed by Tukey’s multiple comparison test. Binding pocket performance is tested with P value noted as follows. *: P ≤ 0.05, **: P ≤ 0.01, ***: P ≤ 0.001, ****: P ≤ 0.0001, ns: not significantly different. Black asterisks signify the row structure is significantly better than the column structure, and vice-versa for red asterisks.
(PDF)

S6 Table Statistical significance of VS performance between B2AR BI-bound binding pockets. One-way ANOVA was performed on mean NSQ_AUC ± S.E.M. for each of the docking experiments, followed by a Tukey multiple comparison test for a) B2AR agonists vs. decoys (Fig 6b) and b) B2AR agonists vs. B2AR inhibitors (Fig 6c). A one-way ANOVA was carried out, followed by Tukey’s multiple comparison test. Binding pocket performance is tested with P value noted as follows. *: P ≤ 0.05, **: P ≤ 0.01, ***: P ≤ 0.001, ****: P ≤ 0.0001, ns: not significantly different. Black asterisks signify the row structure is significantly better than the column structure, and vice-versa for red asterisks.
(PDF)

S7 Table Statistical significance of VS performance between B2AR CAR-bound binding pockets. One-way ANOVA was performed on mean NSQ_AUC ± S.E.M. for each of the docking experiments, followed by a Tukey multiple comparison test for a) B2AR inhibitors vs. decoys (Fig 7b) and b) B2AR inhibitors vs. B2AR agonists (Fig 7c). A one-way ANOVA was carried out, followed by Tukey’s multiple comparison test. Binding pocket performance is tested with P value noted as follows. *: P ≤ 0.05, **: P ≤ 0.01, ***: P ≤ 0.001, ****: P ≤ 0.0001, ns: not significantly different. Black asterisks signify the row structure is significantly better than the column structure, and vice-versa for red asterisks.
(PDF)

S8 Table Statistical significance of VS performance between DOR NAL-bound binding pockets. One-way ANOVA was performed on mean NSQ_AUC ± S.E.M. for each of the docking experiments, followed by a Tukey multiple comparison test for a) DOR inhibitors vs. decoys (Fig 8b) and b) DOR inhibitors vs. DOR agonists (Fig 8c). A one-way ANOVA was carried out, followed by Tukey’s multiple comparison test. Binding pocket performance is tested with P value noted as follows. *: P ≤ 0.05, **: P ≤ 0.01, ***: P ≤ 0.001, ****: P ≤ 0.0001, ns: not significantly different. Black asterisks signify the row structure is significantly better than the column structure, and vice-versa for red asterisks.
(PDF)
