# Supplementary material for: Structural features embedded in G protein-coupled receptor co-crystal structures are key to their success in virtual screening
Source: PLoS One. 2017 Apr 5;12(4):e0174719. doi: 10.1371/journal.pone.0174719 (PMC5381884; doi:10.1371/journal.pone.0174719)

S1 Fig. Comparison of VS performance with and without hydrogen bond optimization. The known ligands vs. decoys and agonist vs. inhibitors (or vice versa) are compared respectively for (a,b) AA2AR-ZM 3EML, (c,d) AA2AR-ZM 4EIY, (e,f) B1AR-DOB 2Y00-A, (g,h) B1AR-CYP 2VT4, (i,j) B1AR-CYP 2YCX, (k,l) B1AR-CYP 2YCY, (m,n) B1AR-CYP 4BVN, (o,p) B2AR-BI 3P0G, (q,r) B2AR-BI 4LDE, (s,t) B2AR-BI 3SN6, (u,v) B2AR-CAR 2RH1 and (w,x) DOR-NAL 4N6H.

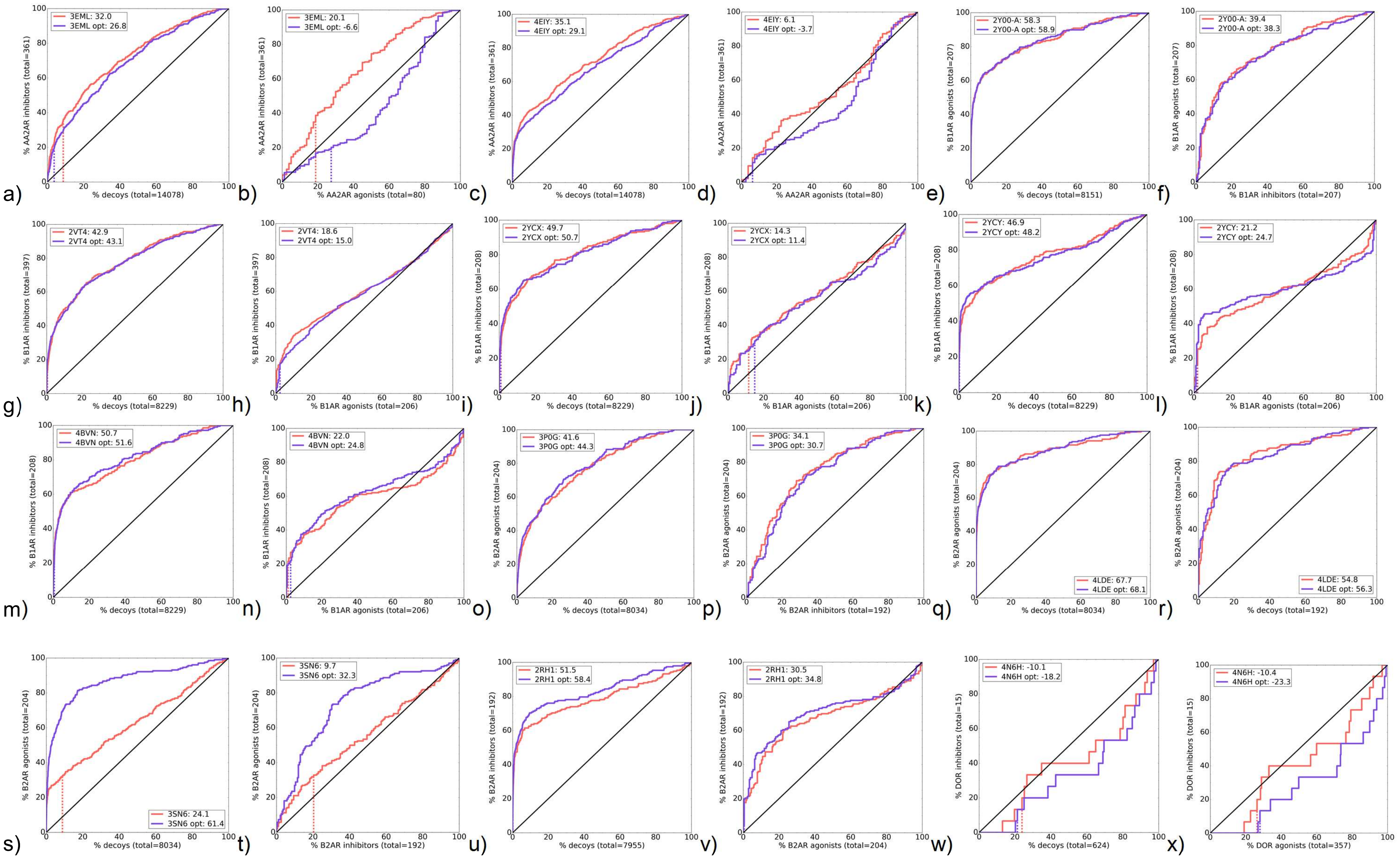

Supplement: S1 Fig — The known ligands vs. decoys and agonist vs. inhibitors (or vice versa) are compared respectively for (a,b) AA2AR-ZM 3EML, (c,d) AA2AR-ZM 4EIY, (e,f) B1AR-DOB 2Y00-A, (g,h) B1AR-CYP 2VT4, (i,j) B1AR-CYP 2YCX, (k,l) B1AR-CYP 2YCY, (m,n) B1AR-CYP 4BVN, (o,p) B2AR-BI 3P0G, (q,r) B2AR-BI 4LDE, (s,t) B2AR-BI 3SN6, (u,v) B2AR-CAR 2RH1 and (w,x) DOR-NAL 4N6H. (PDF) [file pone.0174719.s001.pdf]
