# Supplementary material for: Structural features embedded in G protein-coupled receptor co-crystal structures are key to their success in virtual screening
Source: PLoS One. 2017 Apr 5;12(4):e0174719. doi: 10.1371/journal.pone.0174719 (PMC5381884; doi:10.1371/journal.pone.0174719)

S2 Fig. Ligand library original GLL/GDD vs racemic GLL/GDD NSQ_AUC comparisons. Comparison of VS performance on groups of binding pockets bound by the same ligand depending on the ligand library used for screening: original GLL/GDD (hatched) or racemic GLL/GDD (plain). Values are NSQ_AUCs calculated on ROC curves comparing a) known ligands against decoys, and b) agonists against inhibitors (or vice-versa).

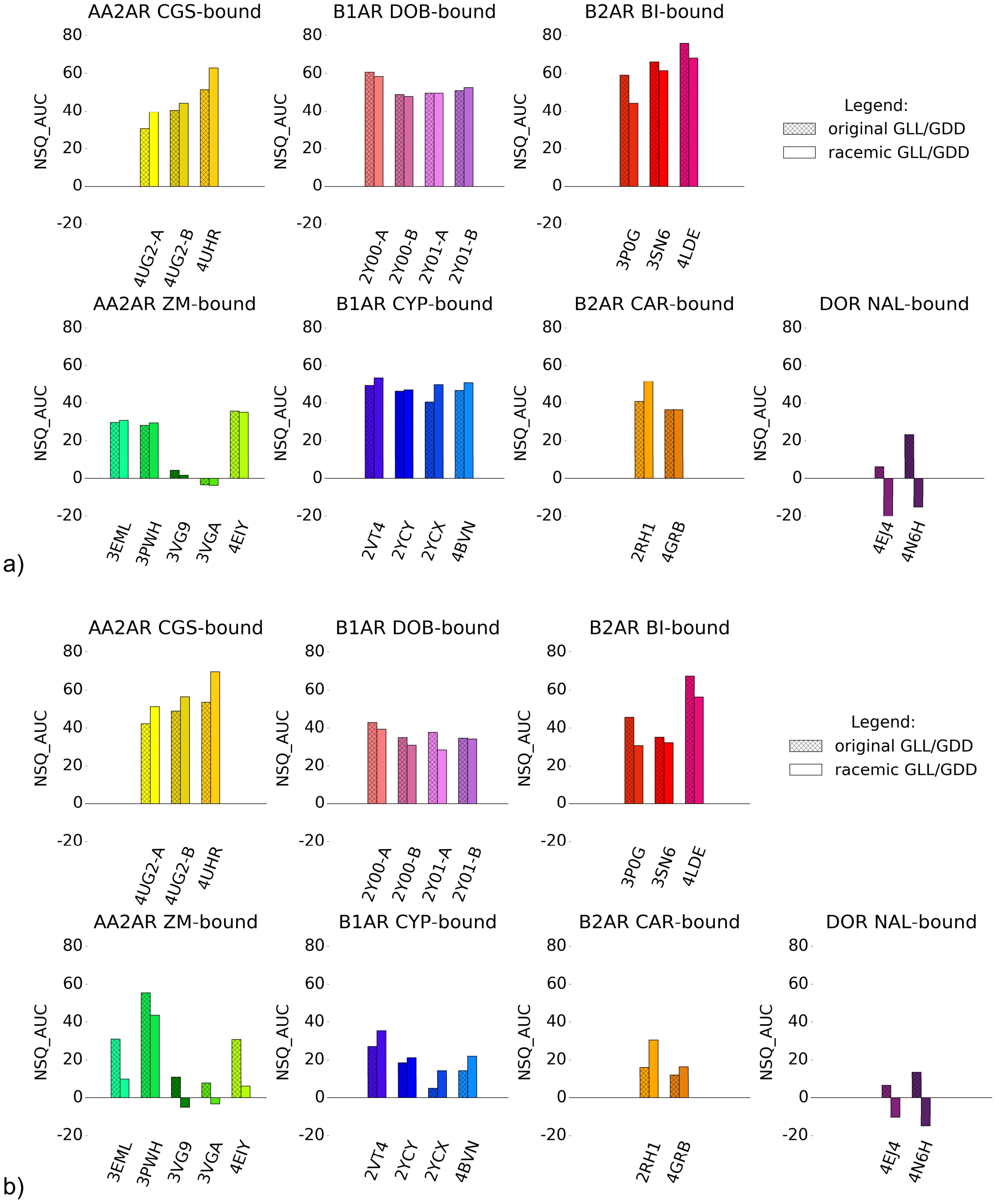

Supplement: S2 Fig — Comparison of VS performance on groups of binding pockets bound by the same ligand depending on the ligand library used for screening: original GLL/GDD (hatched) or racemic GLL/GDD (plain). Values are NSQ_AUCs calculated on ROC curves comparing a) known ligands against decoys, and b) agonists against inhibitors (or vice-versa). (PDF) [file pone.0174719.s002.pdf]
