# Supplementary material for: Structural features embedded in G protein-coupled receptor co-crystal structures are key to their success in virtual screening
Source: PLoS One. 2017 Apr 5;12(4):e0174719. doi: 10.1371/journal.pone.0174719 (PMC5381884; doi:10.1371/journal.pone.0174719)

S5 Fig. Dendrogram of the ligand chemotypes for B2AR. Known a) agonists and b) inhibitors. The number of ligands within each branch is noted. Clusters used in VS are circled and named, and the co-crystal X-ray ligand's position is identified. The asterisk denotes the location of the chemotype cluster center for its respective chemotype cluster, no cluster center is provided for the cluster 'other'.

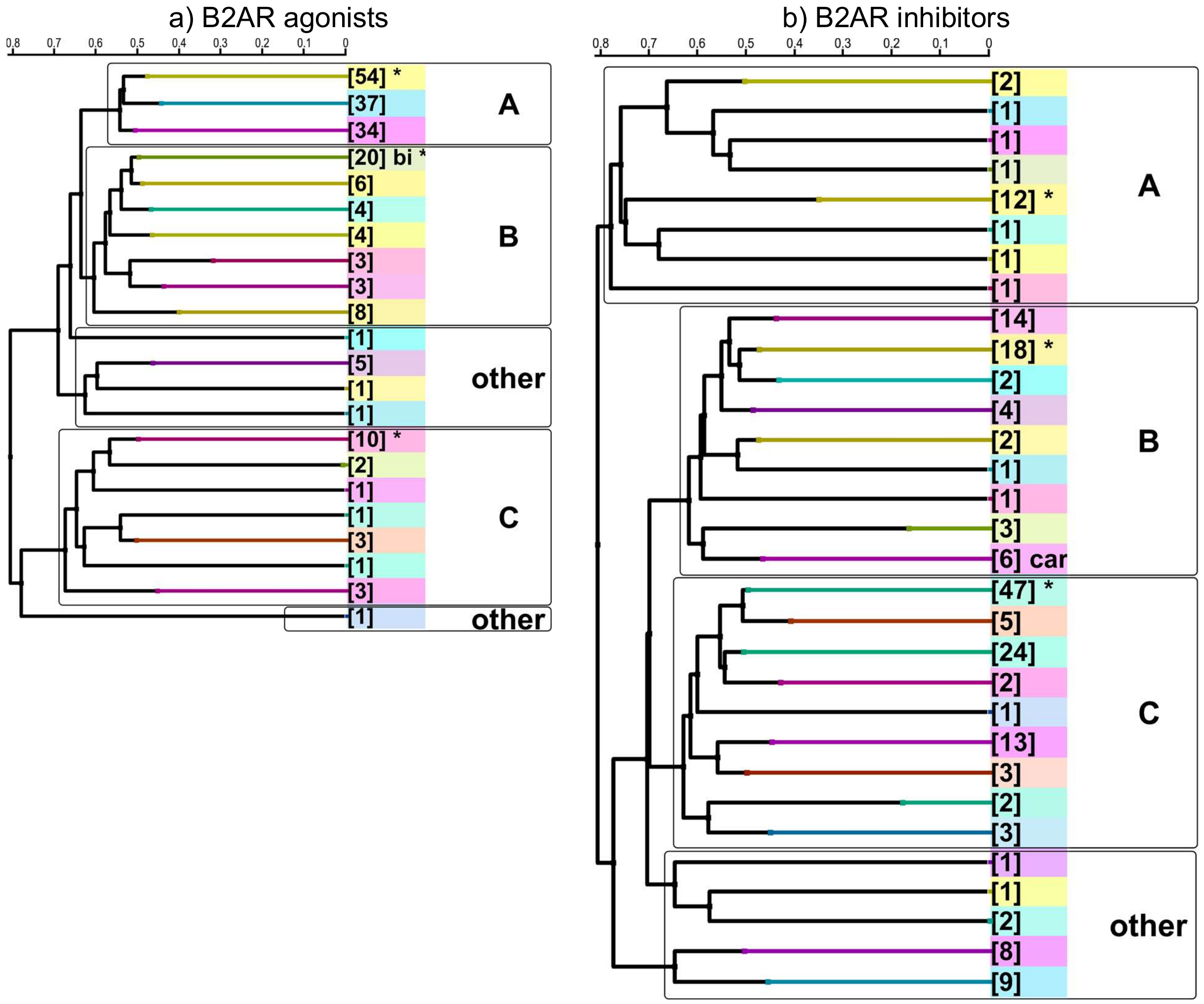

Supplement: S5 Fig — Known a) agonists and b) inhibitors. The number of ligands within each branch is noted. Clusters used in VS are circled and named, and the co-crystal X-ray ligand’s position is identified. The asterisk denotes the location of the chemotype cluster center for its respective chemotype cluster, no cluster center is provided for the cluster ‘other’. (PDF) [file pone.0174719.s005.pdf]
