# Supplementary material for: Structural features embedded in G protein-coupled receptor co-crystal structures are key to their success in virtual screening
Source: PLoS One. 2017 Apr 5;12(4):e0174719. doi: 10.1371/journal.pone.0174719 (PMC5381884; doi:10.1371/journal.pone.0174719)

**S6 Fig. Dendrogram of the ligand chemotypes for DOR.** The number of ligands within each branch is noted. Clusters used in VS are circled and named, and the co-crystal X-ray ligand's position is identified. The asterisk denotes the location of the chemotype cluster center for its respective chemotype cluster, no cluster center is provided for the cluster 'other'.

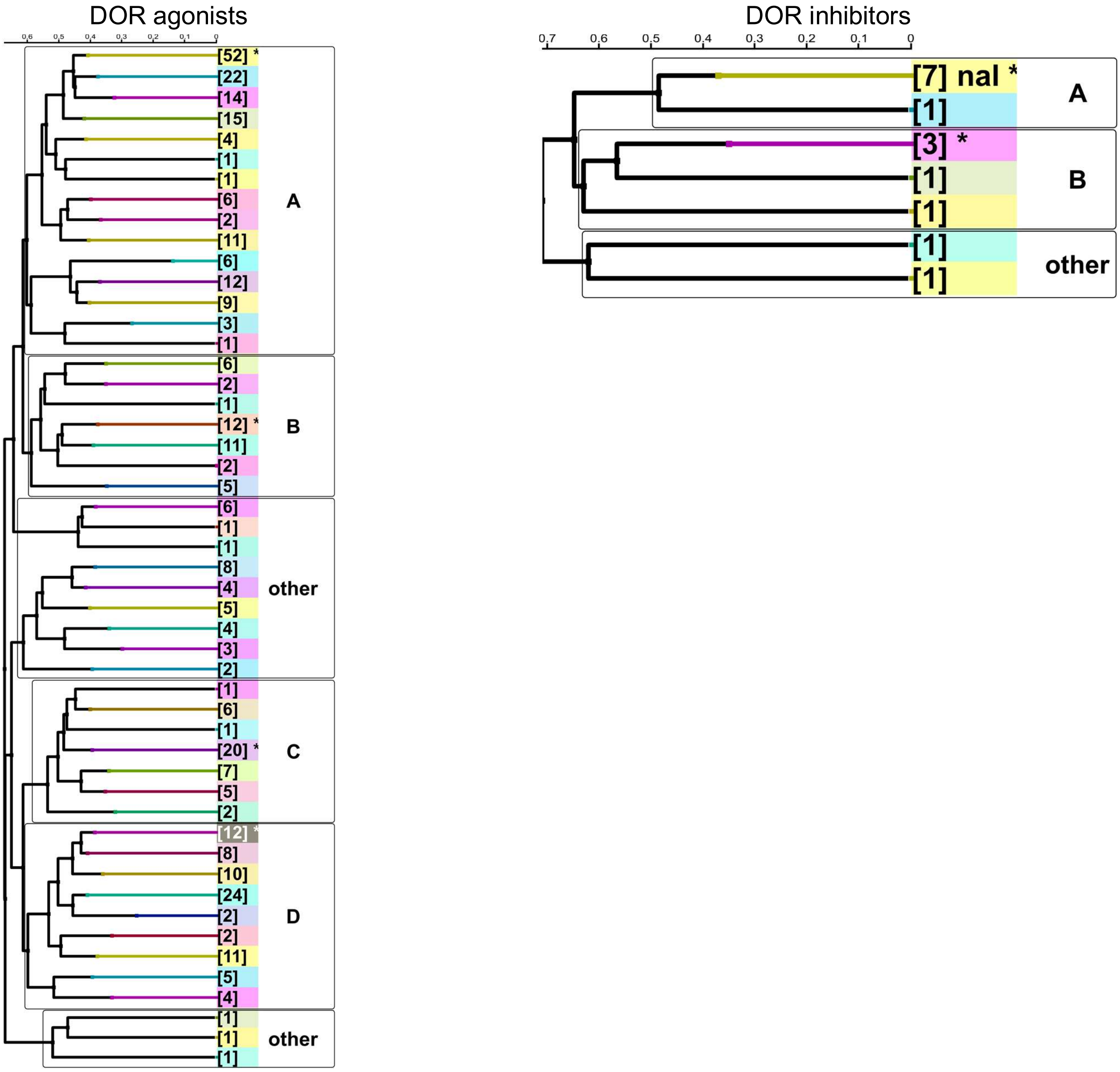

Supplement: S6 Fig — The number of ligands within each branch is noted. Clusters used in VS are circled and named, and the co-crystal X-ray ligand’s position is identified. The asterisk denotes the location of the chemotype cluster center for its respective chemotype cluster, no cluster center is provided for the cluster ‘other’. (PDF) [file pone.0174719.s006.pdf]
