# Supplementary material for: Structural features embedded in G protein-coupled receptor co-crystal structures are key to their success in virtual screening
Source: PLoS One. 2017 Apr 5;12(4):e0174719. doi: 10.1371/journal.pone.0174719 (PMC5381884; doi:10.1371/journal.pone.0174719)

S7 Fig. Enrichment factors of AA2AR known agonist chemotypes for CGS-bound AA2AR binding pockets (4UG2-A, 4UG2-B, 4UHR). Enrichment factors at EF2, EF5 and EF10 using the 2D racemic ligand library.

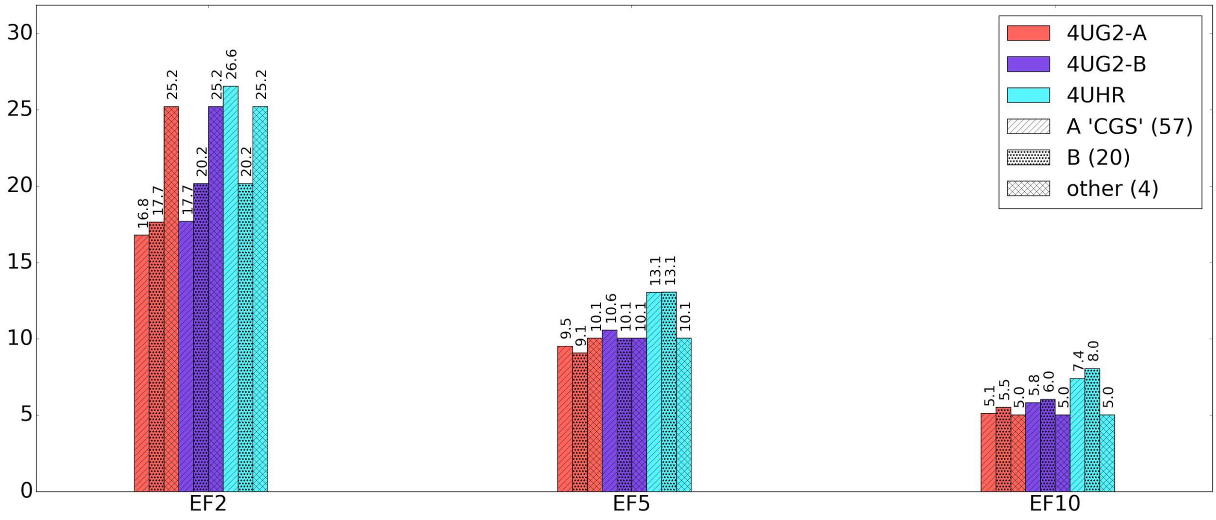

Supplement: S7 Fig — Enrichment factors at EF2, EF5 and EF10 using the 2D racemic ligand library. (PDF) [file pone.0174719.s007.pdf]
