# Supplementary material for: Structural features embedded in G protein-coupled receptor co-crystal structures are key to their success in virtual screening
Source: PLoS One. 2017 Apr 5;12(4):e0174719. doi: 10.1371/journal.pone.0174719 (PMC5381884; doi:10.1371/journal.pone.0174719)

S8 Fig. Cognate docking of the ZM ligand on all AA2AR ZM-bound binding pockets (3EML, 3PWH, 3VG9, 3VGA and 4EIY). All complexes were superimposed and only one representative receptor is displayed in grey, with TM6 and TM7 omitted for clarity. Carbon atoms of the ligands are colored as follows: 3EML (red), 3PWH (purple), 3VG9 (cyan), 3VGA (green), 4EIY (yellow).

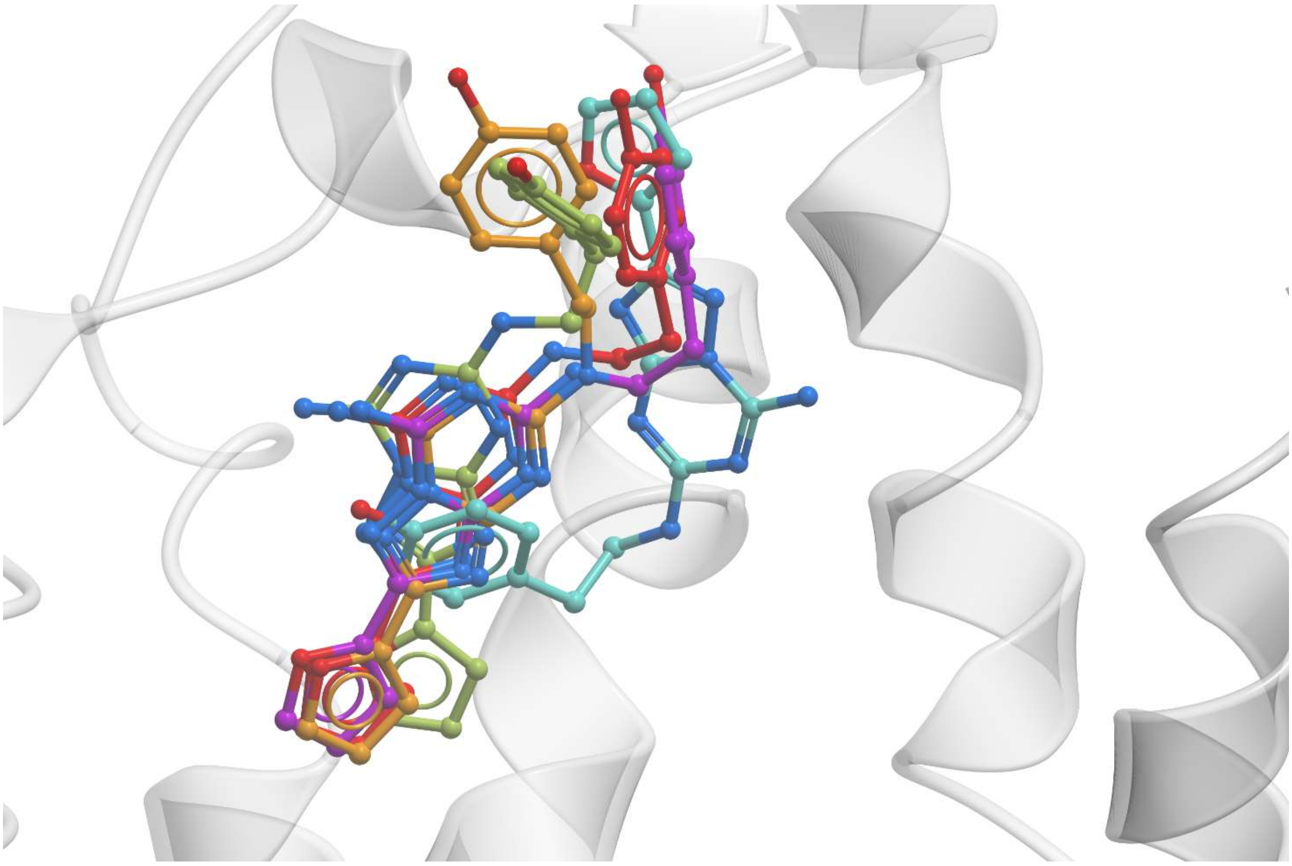

Supplement: S8 Fig — All complexes were superimposed and only one representative receptor is displayed in grey, with TM6 and TM7 omitted for clarity. Carbon atoms of the ligands are colored as follows: 3EML (red), 3PWH (purple), 3VG9 (cyan), 3VGA (green), 4EIY (yellow). (PDF) [file pone.0174719.s008.pdf]
