# Supplementary material for: Structural features embedded in G protein-coupled receptor co-crystal structures are key to their success in virtual screening
Source: PLoS One. 2017 Apr 5;12(4):e0174719. doi: 10.1371/journal.pone.0174719 (PMC5381884; doi:10.1371/journal.pone.0174719)

S9 Fig. Enrichment factors of AA2AR known inhibitor chemotypes for ZM-bound AA2AR binding pockets (3EML, 3PWH, 3VG9, 3VGA, 4EIY). Enrichment factors at EF2, EF5 and EF10.

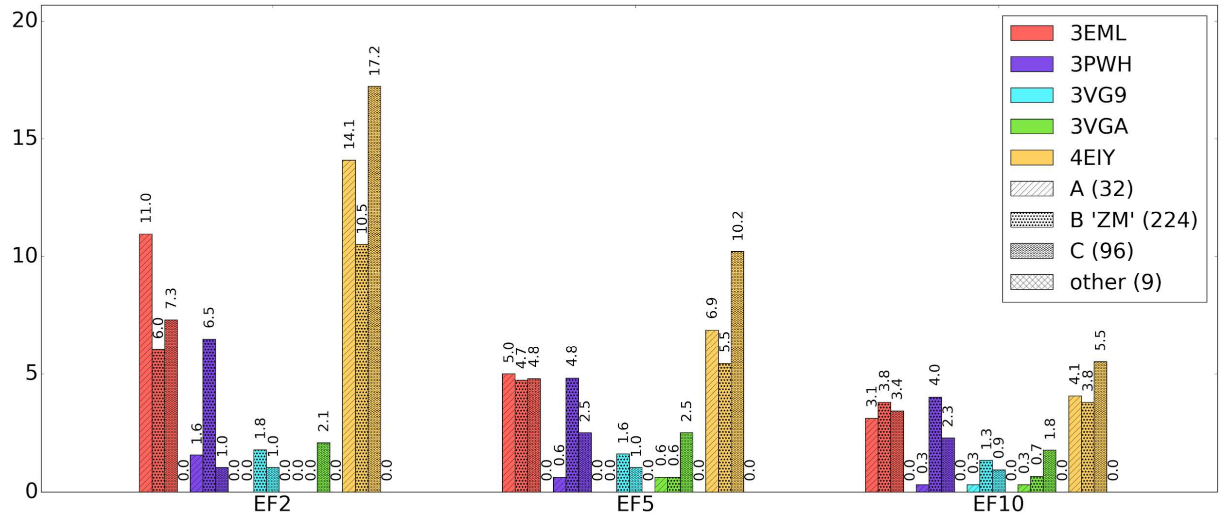

Supplement: S9 Fig — Enrichment factors at EF2, EF5 and EF10. (PDF) [file pone.0174719.s009.pdf]
