# Supplementary material for: Structural features embedded in G protein-coupled receptor co-crystal structures are key to their success in virtual screening
Source: PLoS One. 2017 Apr 5;12(4):e0174719. doi: 10.1371/journal.pone.0174719 (PMC5381884; doi:10.1371/journal.pone.0174719)

S10 Fig. Enrichment factors of B1AR known agonist chemotypes for DOB-bound B1AR binding pockets (2Y00-A, 2Y00-B, 2Y01-A, 2Y01-B). Enrichment factors at EF2, EF5 and EF10.

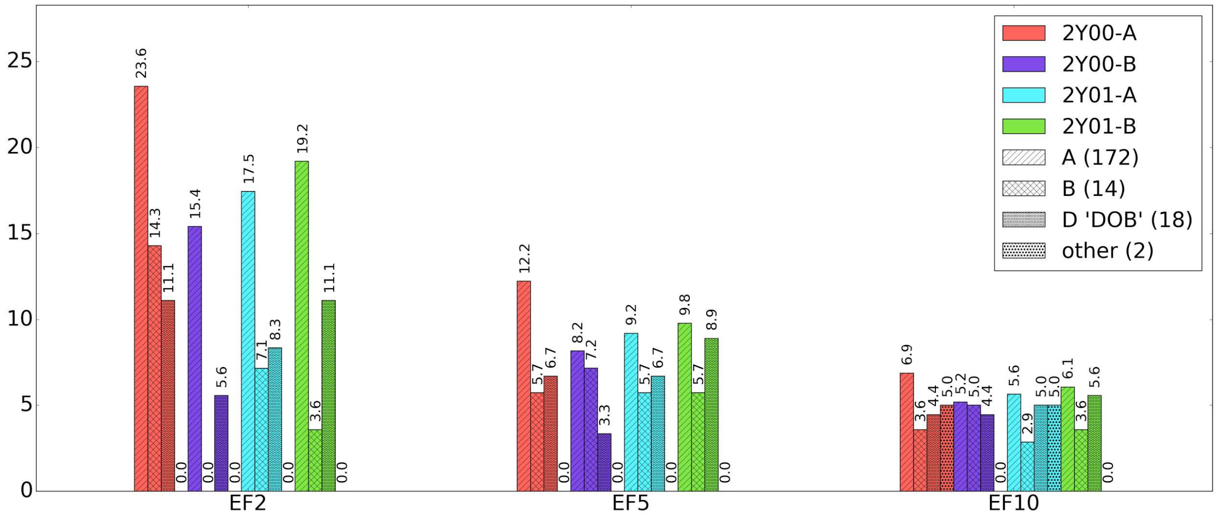

Supplement: S10 Fig — Enrichment factors at EF2, EF5 and EF10. (PDF) [file pone.0174719.s010.pdf]
