# Supplementary material for: Structural features embedded in G protein-coupled receptor co-crystal structures are key to their success in virtual screening
Source: PLoS One. 2017 Apr 5;12(4):e0174719. doi: 10.1371/journal.pone.0174719 (PMC5381884; doi:10.1371/journal.pone.0174719)

S11 Fig: Enrichment factors of B1AR known inhibitor chemotypes for CYP-bound B1AR binding pockets (2VT4, 2YCX, 2YCY, 4BVN). Enrichment factors at EF2, EF5 and EF10.

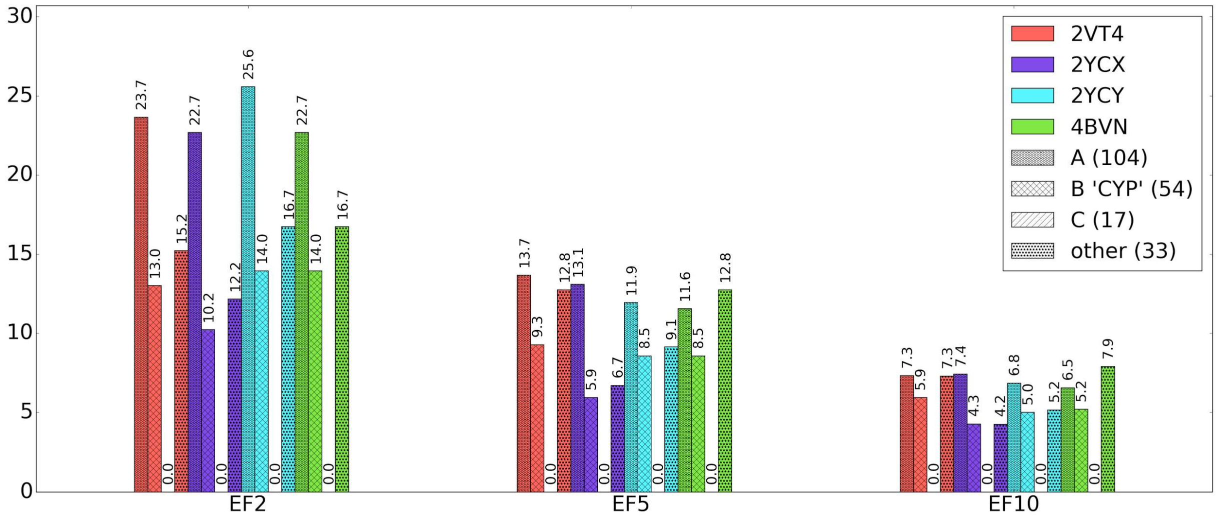

Supplement: S11 Fig — Enrichment factors at EF2, EF5 and EF10. (PDF) [file pone.0174719.s011.pdf]
