# Supplementary material for: Structural features embedded in G protein-coupled receptor co-crystal structures are key to their success in virtual screening
Source: PLoS One. 2017 Apr 5;12(4):e0174719. doi: 10.1371/journal.pone.0174719 (PMC5381884; doi:10.1371/journal.pone.0174719)

S13 Fig. Enrichment factors of B2AR known inhibitor chemotypes for CAR-bound B2AR binding pockets (2RH1, 4GBR). Enrichment factors at EF2, EF5 and EF10.

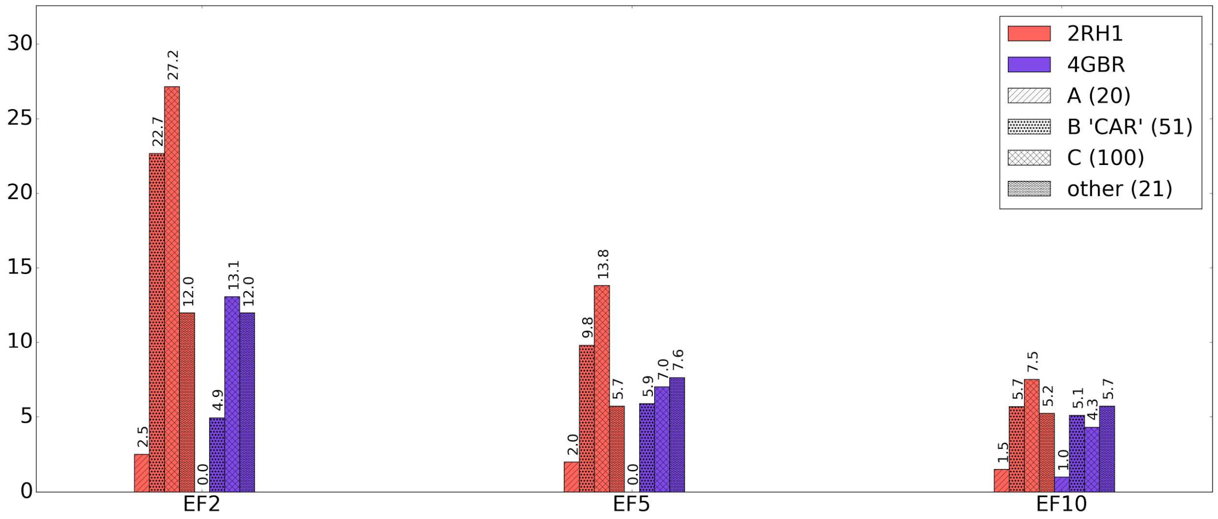

Supplement: S13 Fig — Enrichment factors at EF2, EF5 and EF10. (PDF) [file pone.0174719.s013.pdf]
