# Supplementary material for: Structural features embedded in G protein-coupled receptor co-crystal structures are key to their success in virtual screening
Source: PLoS One. 2017 Apr 5;12(4):e0174719. doi: 10.1371/journal.pone.0174719 (PMC5381884; doi:10.1371/journal.pone.0174719)
Supplement: S15 Fig — Assessment of local model quality for: a) 3EML, b) 3PWH, c) 3VG9, d) 3VGA and e) 4EIY. Real-space correlation coefficient (green) and B-factor values (red) are shown for all residues of the binding pocket and the bound ligand ZM. A green dotted line cutoff value of 0.8 highlights low RSCC values. (PDF) [file pone.0174719.s015.pdf]

S15 Fig. RSCC and B-factor plots for AA2AR ZM-bound binding pockets. Assessment of local model quality for: a) 3EML, b) 3PWH, c) 3VG9, d) 3VGA and e) 4EIY. Real-space correlation coefficient (green) and B-factor values (red) are shown for all residues of the binding pocket and the bound ligand ZM. A green dotted line cutoff value of 0.8 highlights low RSCC values.

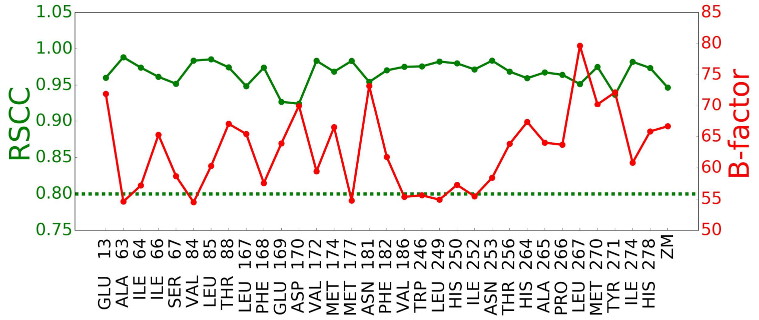

a)

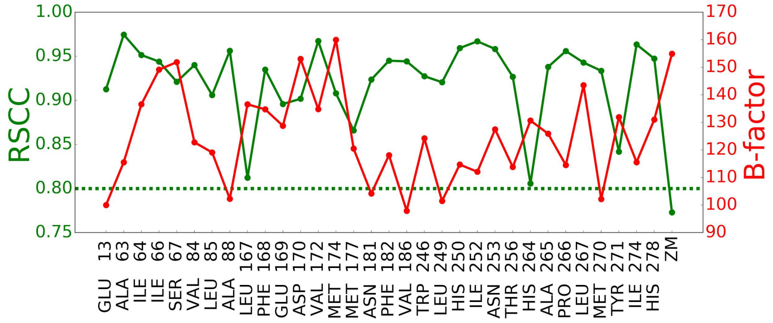

b)

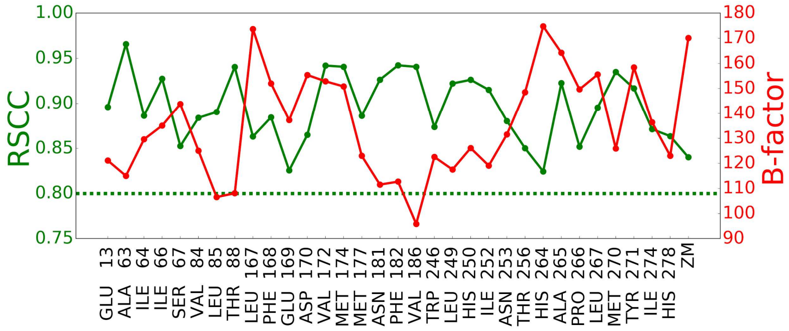

c)

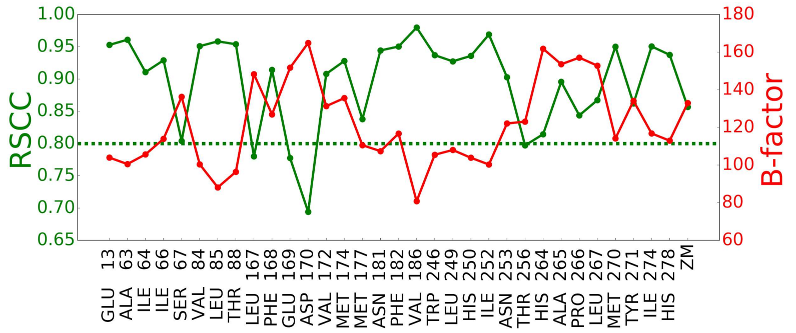
RSCC
B-factor
GLU 13
ALA 63
ILE 64
ILE 66
SER 67
VAL 84
LEU 85
THR 88
LEU 167
PHE 168
GLU 169
ASP 170
VAL 172
MET 174
MET 177
ASN 181
PHE 182
VAL 186
TRP 246
LEU 249
HIS 250
ILE 252
ASN 253
THR 256
HIS 264
ALA 265
PRO 266
LEU 267
MET 270
TYR 271
ILE 274
HIS 278
ZM

d)

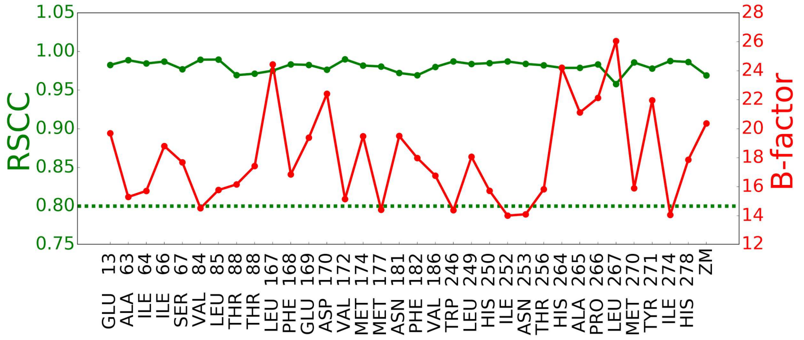
RSCC
B-factor
GLU 13
ALA 63
ILE 64
ILE 66
SER 67
VAL 84
LEU 85
THR 88
THR 88
LEU 167
PHE 168
GLU 169
ASP 170
VAL 172
MET 174
MET 177
ASN 181
PHE 182
VAL 186
TRP 246
LEU 249
HIS 250
ILE 252
ASN 253
THR 256
HIS 264
ALA 265
PRO 266
LEU 267
MET 270
TYR 271
ILE 274
HIS 278
ZM

e)
