# Supplementary material for: Structural features embedded in G protein-coupled receptor co-crystal structures are key to their success in virtual screening
Source: PLoS One. 2017 Apr 5;12(4):e0174719. doi: 10.1371/journal.pone.0174719 (PMC5381884; doi:10.1371/journal.pone.0174719)

S16 Fig. RSCC and B-factor plots for B1AR DOB-bound binding pockets. Assessment of local model quality for: a) 2Y00-A, b) 2Y00-B, c) 2Y01-A and d) 2Y01-B. Real-space correlation coefficient (green) and B-factor values (red) are shown for all residues of the binding pocket and the bound ligand DOB. A green dotted line cutoff value of 0.8 highlights low RSCC values.

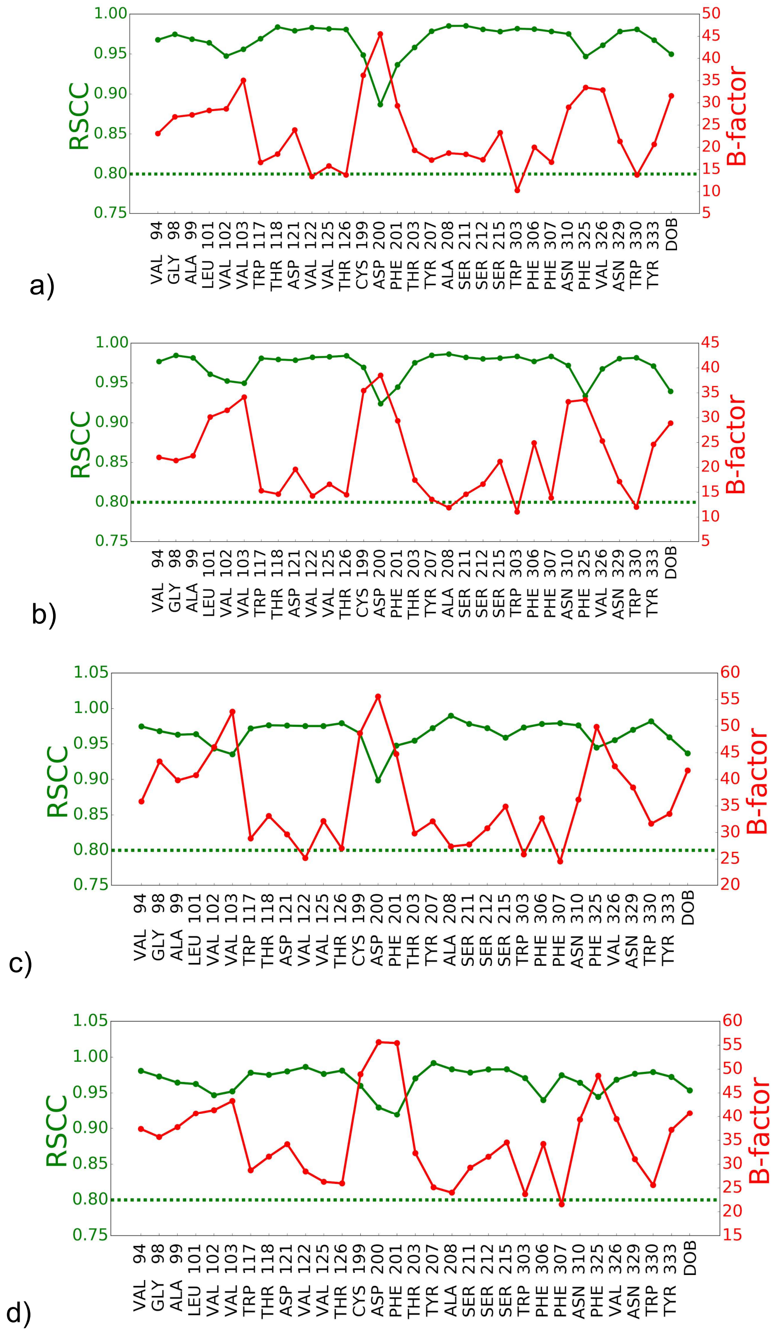

Supplement: S16 Fig — Assessment of local model quality for: a) 2Y00-A, b) 2Y00-B, c) 2Y01-A and d) 2Y01-B. Real-space correlation coefficient (green) and B-factor values (red) are shown for all residues of the binding pocket and the bound ligand DOB. A green dotted line cutoff value of 0.8 highlights low RSCC values. (PDF) [file pone.0174719.s016.pdf]
