# Supplementary material for: Structural features embedded in G protein-coupled receptor co-crystal structures are key to their success in virtual screening
Source: PLoS One. 2017 Apr 5;12(4):e0174719. doi: 10.1371/journal.pone.0174719 (PMC5381884; doi:10.1371/journal.pone.0174719)
Supplement: S18 Fig — Assessment of local model quality for: a) 3P0G, b) 3SN6 and c) 4LDE. Real-space correlation coefficient (green) and B-factor values (red) are shown for all residues of the binding pocket and the bound ligand BI. A green dotted line cutoff value of 0.8 highlights low RSCC values. (PDF) [file pone.0174719.s018.pdf]

**S18 Fig. RSCC and B-factor plots for B2AR BI-bound binding pockets.** Assessment of local model quality for: a) 3P0G, b) 3SN6 and c) 4LDE. Real-space correlation coefficient (green) and B-factor values (red) are shown for all residues of the binding pocket and the bound ligand BI. A green dotted line cutoff value of 0.8 highlights low RSCC values.

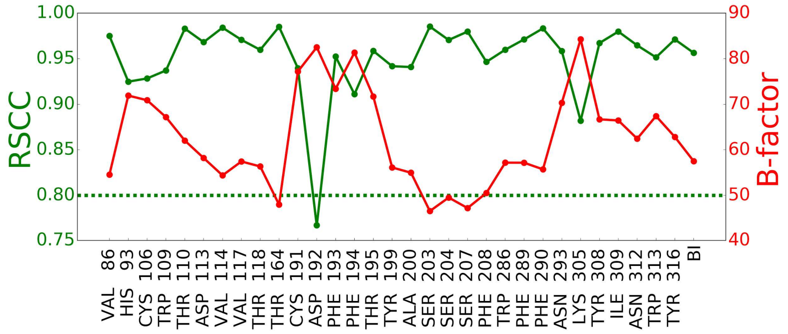


a)

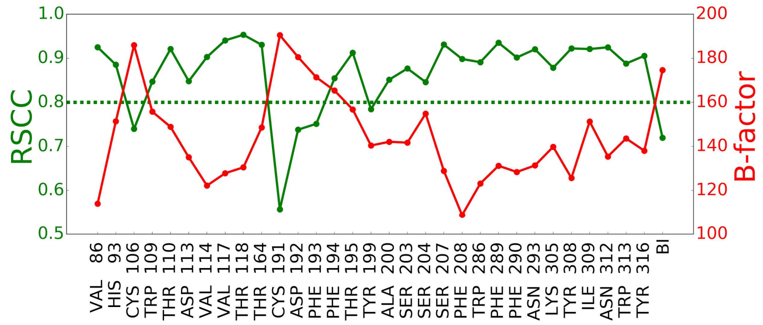


b)

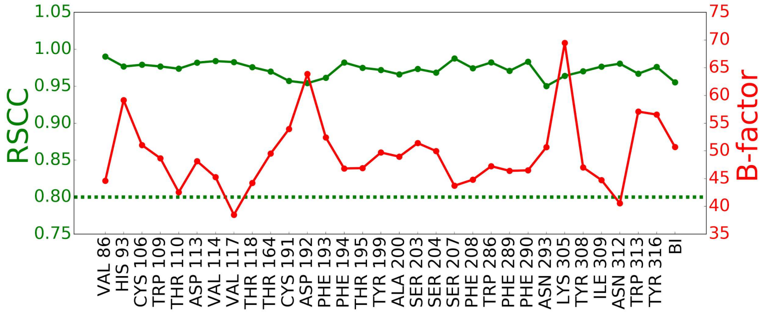


c)
