# Supplementary material for: Structural features embedded in G protein-coupled receptor co-crystal structures are key to their success in virtual screening
Source: PLoS One. 2017 Apr 5;12(4):e0174719. doi: 10.1371/journal.pone.0174719 (PMC5381884; doi:10.1371/journal.pone.0174719)
Supplement: S21 Fig — VS results displayed as ROC curves of (a) DOR inhibitors against decoys and (b) DOR inhibitors against DOR agonists. The ROC curves are representations of the VS, picking the best scoring ligand after docking three independent times. A black line depicts the hypothetical random recovery of true positives. The rank of the docked co-crystal ligand relative to the percentage false positives is identified with a vertical dashed line. The inset values are NSQ_AUCs calculated on these representative curves. (PDF) [file pone.0174719.s021.pdf]

S21 Fig. Analyzing the influence of the docking effort parameter on VS performance for DOR binding pockets (4EJ4 and 4N6H). VS results displayed as ROC curves of (a) DOR inhibitors against decoys and (b) DOR inhibitors against DOR agonists. The ROC curves are representations of the VS, picking the best scoring ligand after docking three independent times. A black line depicts the hypothetical random recovery of true positives. The rank of the docked co-crystal ligand relative to the percentage false positives is identified with a vertical dashed line. The inset values are NSQ_AUCs calculated on these representative curves.

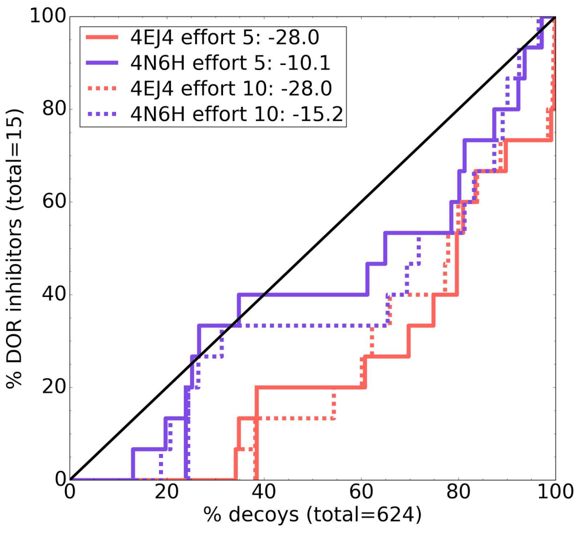

a)

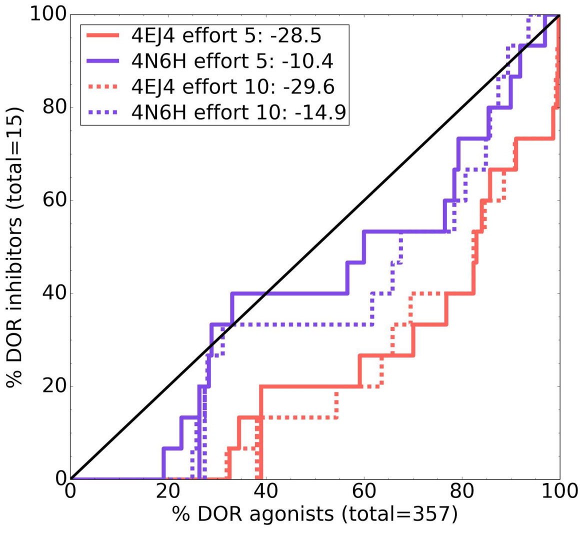

b)
