# Supplementary material for: Structural features embedded in G protein-coupled receptor co-crystal structures are key to their success in virtual screening
Source: PLoS One. 2017 Apr 5;12(4):e0174719. doi: 10.1371/journal.pone.0174719 (PMC5381884; doi:10.1371/journal.pone.0174719)
Supplement: S1 Table — Details about known ligand count, chiral center composition, as well as chemotype clusters names and counts. A chemical structure for the center of each chemotype cluster is represented, as well as each ligand from co-crystal X-ray structures. (PDF) [file pone.0174719.s022.pdf]

S1 Table. Known ligand library and decoys for each GPCR. Details about known ligand count, chiral center composition, as well as chemotype clusters names and counts. A chemical structure for the center of each chemotype cluster is represented, as well as each ligand from co-crystal X-ray structure.

Library name	Known ligand count	Chirality		Chemotype main clusters			Co-crystal ligand	Decoys #
		% of compounds with at least one chiral center	avg. # of chiral center per compound	Name	Count	Center		
AA2AR agonists	81	100.0	4.07	A 'CGS'	57	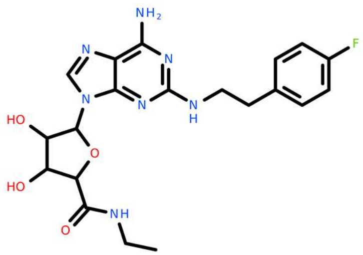	CGS 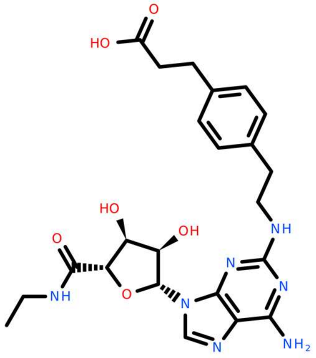	3198

				B	20	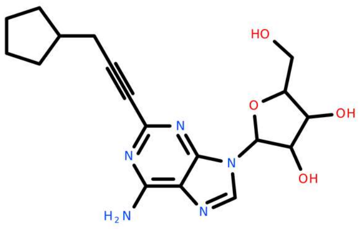		
AA2AR inhibitors	361	14.40	0.26	A	32	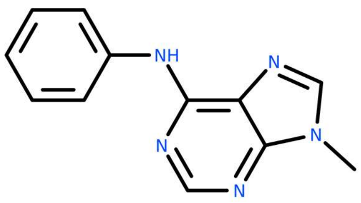	ZM 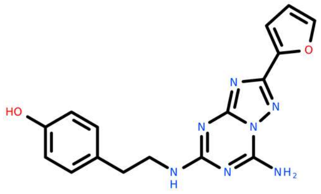	14078
				B 'ZM'	224	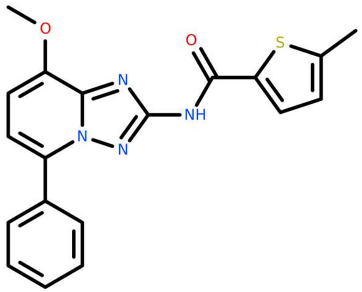		
				C	96	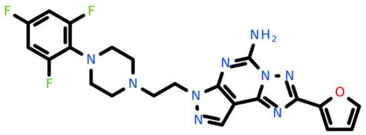		

B1AR agonists	206	81.07	1.33	A	172	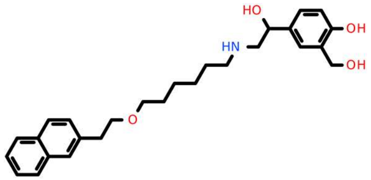	DOB 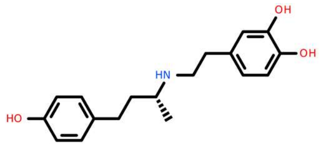	8151
				B	14	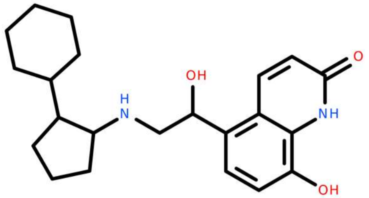		
				C 'DOB'	18	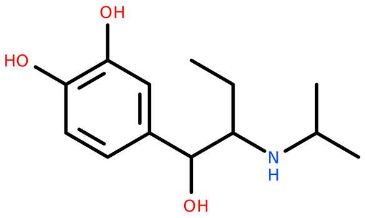		
B1AR inhibitors	208	90.38	1.23	A		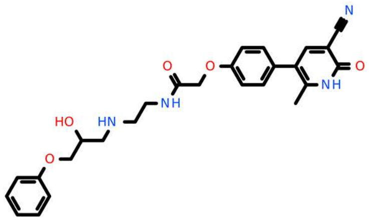	CYP 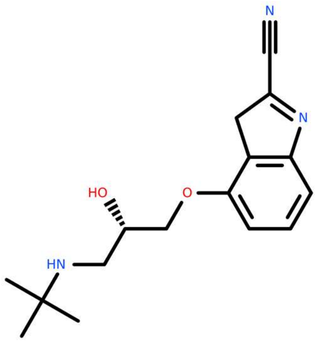	8229

				B ‘CYP’		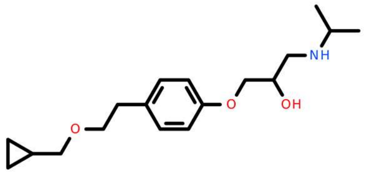		
				C		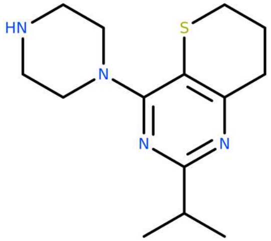		
B2AR agonists	204	81.28	1.35	A	125	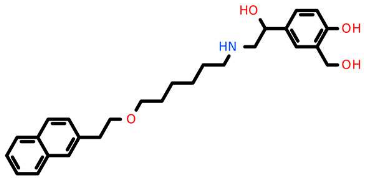	BI 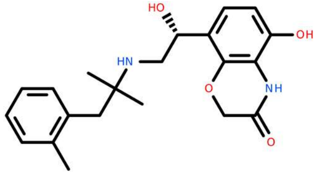	8034
				B ‘BI’	48	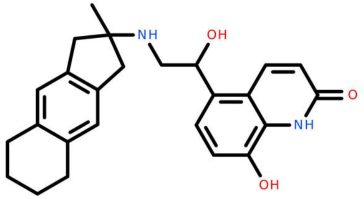		
				C	21	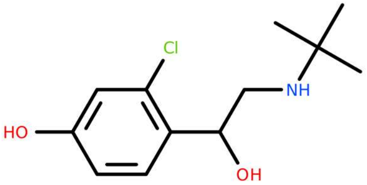		

B2AR inhibitors	192	90.63	1.22	A	20	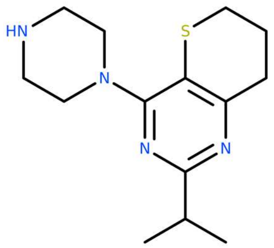	CAR 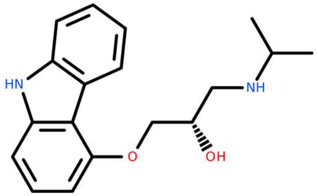	7955
				B ‘CAR’	51	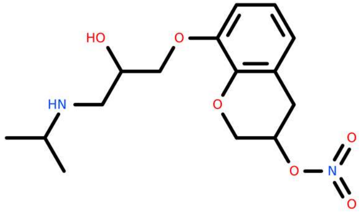		
				C	100	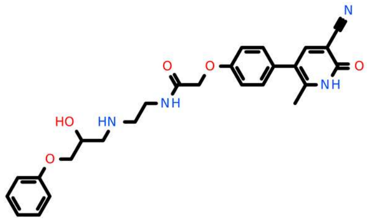		
DOR agonists	357	45.35	1.52	A	165	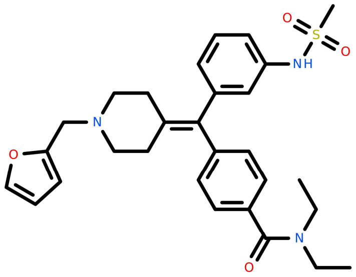		-

				B	39	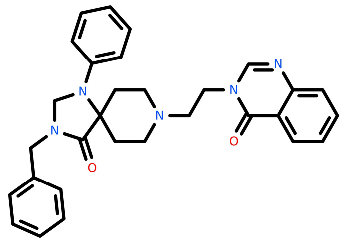		
				C	42	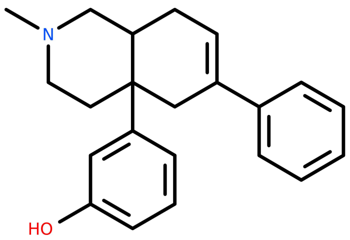		
				D	78	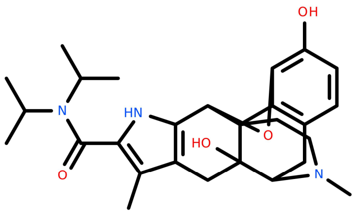		
DOR inhibitors	15	93.33	4.13	A ‘NAL’	8	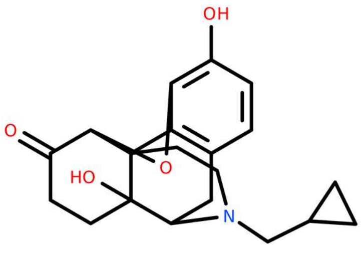	NAL 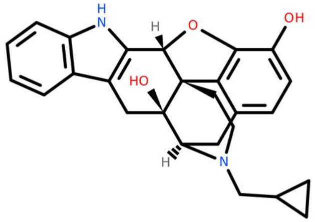	624

				B	5	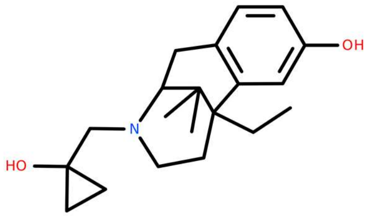		
