# Supplementary material for: Structural features embedded in G protein-coupled receptor co-crystal structures are key to their success in virtual screening
Source: PLoS One. 2017 Apr 5;12(4):e0174719. doi: 10.1371/journal.pone.0174719 (PMC5381884; doi:10.1371/journal.pone.0174719)
Supplement: S2 Table — One-way ANOVA was performed on mean NSQ_AUC ± S.E.M. for each of the docking experiments, followed by a Tukey multiple comparison test for a) AA2AR agonists vs. decoys (Fig 1b) and b) AA2AR agonists vs. AA2AR inhibitors (Fig 1c). A one-way ANOVA was carried out, followed by a Tukey’s multiple comparison test. Binding pocket performance was tested with P value noted as follows. *: P ≤ 0.05, **: P ≤ 0.01, ***: P ≤ 0.001, ****: P ≤ 0.0001, ns: not significantly different. Black asterisks signify the row structure is significantly better than the column structure, and vice-versa for red asterisks. (PDF) [file pone.0174719.s023.pdf]

S2 Table. Statistical significance of VS performance between AA2AR CGS-bound binding pockets. One-way ANOVA was performed on mean NSQ_AUC ± S.E.M. for each of the docking experiments, followed by a Tukey multiple comparison test for a) AA2AR agonists vs. decoys (Fig 1b) and b) AA2AR agonists vs. AA2AR inhibitors (Fig 1c). Binding pocket performance is tested with P value noted as follows. *: P ≤ 0.05, **: P ≤ 0.01, ***: P ≤ 0.001, ****: P ≤ 0.0001, ns: not significantly different. Black asterisks signify the row structure is significantly better than the column structure, and vice-versa for red asterisks.

a) AA2AR agonists vs. decoys

	4UG2-A	4UG2-B	4UHR
4UG2-A		ns	***
4UG2-B			**
4UHR			

b) AA2AR agonists vs. AA2AR inhibitors

	4UG2-A	4UG2-B	4UHR
4UG2-A		*	***
4UG2-B			**
4UHR			
