# Supplementary material for: Structural features embedded in G protein-coupled receptor co-crystal structures are key to their success in virtual screening
Source: PLoS One. 2017 Apr 5;12(4):e0174719. doi: 10.1371/journal.pone.0174719 (PMC5381884; doi:10.1371/journal.pone.0174719)
Supplement: S3 Table — One-way ANOVA was performed on mean NSQ_AUC ± S.E.M. for each of the docking experiments, followed by a Tukey multiple comparison test for a) AA2AR inhibitors vs. decoys (Fig 3b) and b) AA2AR inhibitors vs. AA2AR agonists (Fig 3c). A one-way ANOVA was carried out, followed by Tukey’s multiple comparison test. Binding pocket performance is tested with P value noted as follows. *: P ≤ 0.05, **: P ≤ 0.01, ***: P ≤ 0.001, ****: P ≤ 0.0001, ns: not significantly different. Black asterisks signify the row structure is significantly better than the column structure, and vice-versa for red asterisks. (PDF) [file pone.0174719.s024.pdf]

S3 Table. Statistical significance of VS performance between AA2AR ZM-bound binding pockets. One-way ANOVA was performed on mean NSQ_AUC ± S.E.M. for each of the docking experiments, followed by a Tukey multiple comparison test for a) AA2AR inhibitors vs. decoys (Fig 3b) and b) AA2AR inhibitors vs. AA2AR agonists (Fig 3c). Binding pocket performance is tested with P value noted as follows. *: P ≤ 0.05, **: P ≤ 0.01, ***: P ≤ 0.001, ****: P ≤ 0.0001, ns: not significantly different. Black asterisks signify the row structure is significantly better than the column structure, and vice-versa for red asterisks.

a) AA2AR inhibitors vs. decoys

	3EML	3PWH	3VG9	3VGA	4EIY
3EML		****	****	****	***
3PWH			****	****	****
3VG9				****	****
3VGA					****
4EIY					

b) AA2AR inhibitors vs. agonists

	3EML	3PWH	3VG9	3VGA	4EIY
3EML		****	ns	ns	ns
3PWH			****	****	****
3VG9				ns	ns
3VGA					ns
4EIY					
