# Supplementary material for: Structural features embedded in G protein-coupled receptor co-crystal structures are key to their success in virtual screening
Source: PLoS One. 2017 Apr 5;12(4):e0174719. doi: 10.1371/journal.pone.0174719 (PMC5381884; doi:10.1371/journal.pone.0174719)
Supplement: S4 Table — One-way ANOVA was performed on mean NSQ_AUC ± S.E.M. for each of the docking experiments, followed by a Tukey multiple comparison test for a) B1AR agonists vs. decoys (Fig 4b) and b) B1AR agonists vs. B1AR inhibitors (Fig 4c). A one-way ANOVA was carried out, followed by Tukey’s multiple comparison test. Binding pocket performance is tested with P value noted as follows. *: P ≤ 0.05, **: P ≤ 0.01, ***: P ≤ 0.001, ****: P ≤ 0.0001, ns: not significantly different. Black asterisks signify the row structure is significantly better than the column structure, and vice-versa for red asterisks. (PDF) [file pone.0174719.s025.pdf]

S4 Table. Statistical significance of VS performance between B1AR DOB-bound binding pockets. One-way ANOVA was performed on mean NSQ_AUC ± S.E.M. for each of the docking experiments, followed by a Tukey multiple comparison test for a) B1AR agonists vs. decoys (Fig 4b) and b) B1AR agonists vs. B1AR inhibitors (Fig 4c). Binding pocket performance is tested with P value noted as follows. *: P ≤ 0.05, **: P ≤ 0.01, ***: P ≤ 0.001, ****: P ≤ 0.0001, ns: not significantly different. Black asterisks signify the row structure is significantly better than the column structure, and vice-versa for red asterisks.

a) B1AR agonists vs. decoys

	2Y00-A	2Y00-B	2Y01-A	2Y01-B
2Y00-A		****	****	***
2Y00-B			ns	ns
2Y01-A				ns
2Y01-B				

b) B1AR agonists vs. B1AR inhibitors

	2Y00-A	2Y00-B	2Y01-A	2Y01-B
2Y00-A		*	**	*
2Y00-B			ns	ns
2Y01-A				ns
2Y01-B				
