# Supplementary material for: Structural features embedded in G protein-coupled receptor co-crystal structures are key to their success in virtual screening
Source: PLoS One. 2017 Apr 5;12(4):e0174719. doi: 10.1371/journal.pone.0174719 (PMC5381884; doi:10.1371/journal.pone.0174719)
Supplement: S8 Table — One-way ANOVA was performed on mean NSQ_AUC ± S.E.M. for each of the docking experiments, followed by a Tukey multiple comparison test for a) DOR inhibitors vs. decoys (Fig 8b) and b) DOR inhibitors vs. DOR agonists (Fig 8c). A one-way ANOVA was carried out, followed by Tukey’s multiple comparison test. Binding pocket performance is tested with P value noted as follows. *: P ≤ 0.05, **: P ≤ 0.01, ***: P ≤ 0.001, ****: P ≤ 0.0001, ns: not significantly different. Black asterisks signify the row structure is significantly better than the column structure, and vice-versa for red asterisks. (PDF) [file pone.0174719.s029.pdf]

S8 Table. Statistical significance of VS performance between DOR NAL-bound binding pockets. An unpaired t test with Welch's correction was carried out on mean NSQ_AUC ± S.E.M. for each of the docking experiments for a) DOR inhibitors vs. decoys (Fig 8b) and b) DOR inhibitors vs. DOR agonists (Fig 8c). Binding pocket performance is tested with P value noted as follows. *: P ≤ 0.05, **: P ≤ 0.01, ***: P ≤ 0.001, ****: P ≤ 0.0001, ns: not significantly different. Black asterisks signify the row structure is significantly better than the column structure, and vice-versa for red asterisks.

a) DOR inhibitors vs. decoys

	4EJ4	4N6H
4EJ4		**
4N6H		

b) DOR inhibitors vs. DOR agonists

	4EJ4	4N6H
4EJ4		**
4N6H		
