# Supplementary material for: Structural features embedded in G protein-coupled receptor co-crystal structures are key to their success in virtual screening
Source: PLoS One. 2017 Apr 5;12(4):e0174719. doi: 10.1371/journal.pone.0174719 (PMC5381884; doi:10.1371/journal.pone.0174719)
Supplement: S7 Table — One-way ANOVA was performed on mean NSQ_AUC ± S.E.M. for each of the docking experiments, followed by a Tukey multiple comparison test for a) B2AR inhibitors vs. decoys (Fig 7b) and b) B2AR inhibitors vs. B2AR agonists (Fig 7c). A one-way ANOVA was carried out, followed by Tukey’s multiple comparison test. Binding pocket performance is tested with P value noted as follows. *: P ≤ 0.05, **: P ≤ 0.01, ***: P ≤ 0.001, ****: P ≤ 0.0001, ns: not significantly different. Black asterisks signify the row structure is significantly better than the column structure, and vice-versa for red asterisks. (PDF) [file pone.0174719.s028.pdf]

**S7 Table. Statistical significance of VS performance between B2AR CAR-bound binding pockets.** An unpaired t test with Welch's correction was carried out on mean NSQ_AUC ± S.E.M. for each of the docking experiments for a) B2AR inhibitors vs. decoys (Fig 7b) and b) B2AR inhibitors vs. B2AR agonists (Fig 7c). Binding pocket performance is tested with P value noted as follows. *: P ≤ 0.05, **: P ≤ 0.01, ***: P ≤ 0.001, ****: P ≤ 0.0001, ns: not significantly different. Black asterisks signify the row structure is significantly better than the column structure, and vice-versa for red asterisks.

a) B2AR inhibitors vs. decoys

| | 2RH1 | 4GBR |
|---|---|---|
| 2RH1 | | *** |
| 4GBR | | |

b) B2AR inhibitors vs. B2AR agonists

| | 2RH1 | 4GBR |
|---|---|---|
| 2RH1 | | ** |
| 4GBR | | |
